# Optimized Cultivation and Syntrophic Relationship of Anaerobic Benzene-Degrading Enrichment Cultures under Methanogenic Conditions

**DOI:** 10.1264/jsme2.ME21028

**Published:** 2021-08-24

**Authors:** Hop V. Phan, Futoshi Kurisu, Koichiro Kiba, Hiroaki Furumai

**Affiliations:** 1 JSPS International Research Fellow, Research Center for Water Environment Technology, The University of Tokyo, 7–3–1, Hongo, Bunkyo, Tokyo 113–8656, Japan; 2 Research Center for Water Environment Technology, The University of Tokyo, 7–3–1, Hongo, Bunkyo, Tokyo 113–8656, Japan; 3 Department of Urban Engineering, Graduate School of Engineering, The University of Tokyo, 7–3–1, Hongo, Bunkyo, Tokyo 113–8656, Japan

**Keywords:** benzene degrader, anoxic medium, methanogenic enrichment culture, syntrophic relationship, bioremediation

## Abstract

Current challenges in the anaerobic bioremediation of benzene are the lack of capable cultures and limited knowledge on the biodegradation pathway. Under methanogenic conditions, benzene may be mineralized by syntrophic interactions between microorganisms, which are poorly understood. The present study developed an optimized formula for anoxic medium to successfully promote the growth of the putative benzene degrader *Deltaproteobacterium* Hasda-A and enhance the benzene degradation activity of methanogenic enrichment cultures. Within 70‍ ‍d of incubation, the benzene degradation activity and relative abundance of Hasda-A in cultures in the new defined medium increased from 0.5 to >3‍ ‍mg L^–1^ d^–1^ and from 2.5% to >17%, respectively. Together with Hasda-A, we found a strong positive relationship between the abundances of superphylum OD1 bacteria, three methanogens (*Methanoregula*, *Methanolinea*, and *Methanosaeta*) and benzene degradation activity. The syntrophic relationship between these microbial taxa and Hasda-A was then demonstrated in a correlation analysis of longitudinal data. The involvement of methanogenesis in anaerobic benzene mineralization was confirmed by inhibition experiments. The high benzene degradation activity and growth of Hasda-A were quickly recovered in successive dilutions of enrichment cultures, proving the feasibility of using the medium developed in the present study to produce highly capable cultures. The present results will facilitate practical applications in bioremediation and research on the molecular mechanisms underlying benzene activation and syntrophic interactions in benzene mineralization.

Benzene is a prevalent organic pollutant in contaminated aquifers, mainly from the petroleum industry. Benzene contamination is a source of concern because of its toxicity, carcinogenicity, and solubility. Thus, stringent environmental standards have been imposed (0.01‍ ‍mg L^–1^ in Japan, 0.005‍ ‍mg L^–1^ in the USA, and 0.5‍ ‍mg L^–1^ in EU). The microbial degradation of benzene occurs readily under aerobic conditions, but slowly under anaerobic conditions with a half-life of approximately 210‍ ‍d (Lawrence, 2006).

Some understanding of anaerobic benzene degradation has been achieved, but mainly under nitrate- and iron-reducing conditions. Several pure cultures of anaerobic benzene-degrading microorganisms were obtained under nitrate-reducing (*Decholoromonas* and *Azoarcus* strains) (Coates *et al.*, 2001; Kasai *et al.*, 2006) and iron-reducing conditions (*Ferroglobus placidus* and *Geobacter* strains) (Holmes *et al.*, 2011; Zhang *et al.*, 2012). Although enrichment cultures have been described (Musat and Widdel, 2008; Abu Laban *et al.*, 2009; Noguchi *et al.*, 2014; Luo *et al.*, 2016), no isolates have been obtained that degrade benzene under sulfate-reducing or methanogenic conditions. The biochemical pathway of anaerobic benzene degradation remains elusive, particularly under sulfate-reducing and methanogenic conditions.

Due to the quick depletion of energetically favorable electron acceptors and limited mass transfer in contaminated aquifers, methanogenic biodegradation is considered to play a major role in the microbial clean-up of organic compounds in aquifers (Meckenstock *et al.*, 2015). In terms of anaerobic benzene removal, benzene-degrading consortia have rarely been reported under methanogenic conditions. This may be because of the extremely slow growth rate of microorganisms under methanogenic conditions. Nearly two-decade enrichment cultures yielded a putative benzene degrader (belonging to *Deltaproteobacteria*, designated as ORM2) under methanogenic conditions with a doubling time of more than 30‍ ‍d (Ulrich and Edwards, 2003; Luo *et al.*, 2016). Using DNA stable isotope probing, our colleagues characterized the putative benzene degrader, designated as *Deltaproteobacterium* Hasda-A (Sakai *et al.*, 2009; Noguchi *et al.*, 2014). This microorganism shares a nearly identical 16S rRNA gene sequence with *Deltaproteobacterium* ORM2. The complete mineralization of benzene to methane and carbon dioxide by our methanogenic enrichment cultures was proven by Masumoto *et al.* (2012b) using a ^13^C-labeled substrate. Our benzene-degrading enrichment cultures may also effectively mineralize toluene, benzoate, and phenol (Masumoto *et al.*, 2012b).

Despite extensive efforts, the cultivation of methanogenic benzene-degrading enrichment cultures is still challenging. The enrichment consortia had slow growth rates with limited growth yields. The transfer of active cultures into a new medium typically led to long lag periods and unstable degradation activity that may not be recovered (Sakai *et al.*, 2009; Luo, 2016). These factors represent a substantial bottleneck for studying the molecular mechanisms of biochemical reactions as well as for scaling up the culture volume for the bioremediation of polluted environments. Therefore, it is critical to optimize the cultivation of benzene-degrading cultures under methanogenic conditions. Previous attempts were conducted by our colleagues using different cultivation strategies. Masumoto *et al.* (2012a) reported higher benzene degradation rates at cultivation temperatures of 25–31°C than at higher temperatures. The addition of some organic acids retarded degradation activity (Masumoto *et al.*, 2012a). Similar studies were conducted by another research group using different mineral media, trace mineral components, and reductants (Luo, 2016). However, major improvements have not yet been achieved.

The difficulties associated with cultivating benzene-degrading microorganisms under methanogenic conditions may be attributed to the likely existence of a syntrophic relationship between microorganisms. Previous studies reported the dominance of a microbial consortium, including fermentative bacteria, acetoclastic methanogens, and hydrogenotrophic methanogens, in methanogenic benzene-degrading enrichment cultures (Sakai *et al.*, 2009; Noguchi *et al.*, 2014; Luo *et al.*, 2016). As documented in previous studies, benzene degradation appears to be initiated by fermentative bacteria. Based on our current understanding of the substrates utilized by methanogens (Evans *et al.*, 2019), they more likely consume the fermentation products rather than activate benzene. Despite these microbial data and hypothesis, there is currently not study demonstrating the syntrophic relationship in methanogenic benzene-degrading cultures.

Therefore, the present study attempted to optimize the growth of benzene degraders, enhance degradation activity, and shorten the lag phase for subculturing using different media. The results obtained are promising for the production of highly capable cultures for use in the molecular study of biochemical reactions and bioremediation. By taking advantage of longitudinal data collected in our laboratory, we estimated the syntrophic relationship of benzene-degrading microorganisms by a correlation analysis.

## Materials and Methods

### Selection and preparation of anoxic medium

To select a defined formula for synthetic medium for methanogenic benzene-degrading cultures, we screened the culture media of bacterial isolates phylogenetically related to putative benzene-degrading *Deltaproteobacterium* Hasda-A (based on similarities in 16S rRNA gene sequences [Accession number: AB291810] [Sakai *et al.*, 2009]) and bacterial isolates capable of degrading other aromatic hydrocarbons (benzoate, phenol, and toluene) under anaerobic conditions. Among the 12 media identified (Text S1), the main components (KH_2_PO_4_, NH_4_Cl, MgCl_2_.6H_2_O, and CaCl_2_.2H_2_O) were the same in all cases and thus were selected for use in our synthetic medium. The concentrations of these salts were defined based on the majority. For example, 0.15‍ ‍g L^–1^ CaCl_2_.2H_2_O was used in eight media; therefore, this concentration was selected for our medium. An exception is MgCl_2_.6H_2_O, the concentration of which significantly varied from 0.1 to 3‍ ‍g L^–1^ in the media surveyed. Therefore, although 0.2‍ ‍g L^–1^ MgCl_2_.6H_2_O was initially selected for our medium, various concentrations of this component were tested. Most of the media used sodium sulfide (0.3‍ ‍g L^–1^) as the sole reductant, while four media included cysteine-HCl (0.3‍ ‍g L^–1^). Since cysteine-HCl may be used by microorganisms as an alternative growth substrate (Toth, 2017), it was eliminated. NaHCO_3_ (2.5‍ ‍g L^–1^) and Na-resazurin (0.001‍ ‍g L^–1^) were the buffering agent and redox indicator, respectively. The same mixture of 10 vitamins was deployed in all media, while the trace element mixture was selected based on the majority. Some components (organic acids, yeast extract, and NaCl) were specific to some media. These compounds were not included in our base medium, but were added separately to investigate their effects on benzene degradation activity. Various concentrations of NaCl, namely, 0.1, 0.5, 1.0, and 5‍ ‍g L^–1^, were tested. Previous studies suggested carboxylation as a mechanism for benzene activation under nitrate-, iron-, and sulfate-reducing conditions (Abu Laban *et al.*, 2010; Holmes *et al.*, 2011; Luo *et al.*, 2014). Riboflavin and prenol are precursors of flavin mononucleotide and dimethylallyl monophosphate, respectively, which are co-factors of carboxylation (Wang *et al.*, 2018). Therefore, a higher concentration of riboflavin (100-fold higher than the concentration in the defined medium) with and without the presence of prenol was examined to establish whether these conditions stimulate benzene degradation activity. The default formulation of and preparation guide for the synthetic medium are shown in Table S1. The components and concentrations evaluated are included in Table S2. Media and solutions were prepared under strictly anoxic conditions following the principles and techniques described elsewhere (Widdel, 2017; Laso-Pérez *et al.*, 2018).

### Experimental culture set-up

An enrichment culture exhibiting benzene degradation activity of ~0.5‍ ‍mg L^–1^ d^–1^ in our laboratory was selected as the seed culture. It has been maintained in Milli-Q water with Na_2_S.9H_2_O (0.3‍ ‍g L^–1^) and L-cysteine (0.3‍ ‍g L^–1^) as reducing agents by replenishing benzene as the only carbon source whenever it is depleted. Cultures were set up anoxically in duplicate by diluting the seed culture three times with the synthetic medium (10‍ ‍mL culture with 20‍ ‍mL medium) in 72-mL glass vials. Culture vials were sealed with Teflon-coated butyl rubber stoppers and aluminum crimp caps (Maruemu). All culture set-ups were performed inside a vinyl anaerobic chamber (Coy Lab) under an N_2_ atmosphere with 1–2% (v/v) H_2_. The headspaces of the culture vials were flushed with a gas mix (80% N_2_: 20% CO_2_ [v/v]) to remove H_2_ and a slightly positive pressure was maintained to prevent infiltration by air. Duplicate sterilized cultures were included as negative controls. Cultures were incubated statically at 25°C in the upside-down position to avoid any potential intrusion of air from the outside by blocking the stopper by the media. Benzene was injected using gastight syringes from anoxic benzene-saturated solution. This solution was prepared by overlaying benzene on oxygen-free Milli-Q water that contained Na_2_S.9H_2_O (0.3‍ ‍g L^–1^) as a reducing agent and Na-resazurin (0.001‍ ‍g L^–1^) as an oxygen indicator. Successful enrichment cultures were successively diluted with the defined medium. All subsequent diluted cultures were prepared as 200‍ ‍mL culture in 500-mL vials, leaving a headspace of 300‍ ‍mL. All cultures had a final pH of 6.9–7.2.

### Microbial community study

Biomass samples were collected on day 68 for microbial study. Twelve cultures were selected representing high, medium, and low degradation activities. The seed culture was also included. Samples were centrifuged at 14,000×*g* for 3‍ ‍min to pellet cells. DNA extraction was performed using a FastDNA SPIN Kit for Soil and the FastPrep Instrument (MP Biomedicals). The DNA extract was then purified using the Promega Wizard DNA clean-up system (Promega). DNA quality was checked by 1% agarose gel electrophoresis and using a NanoDrop 2000 spectrophotometer (Thermo Scientific). DNA quantification was performed using the Qubit dsDNA HS Assay Kit (Invitrogen). Bacterial and archaeal 16S rRNA V4 regions were both amplified using the primer pair 515F (5′-GTGCCAGCMGCCGCGGTAA-3′) and 806R (5′-GGACTACHVGGGTWTCTAAT-3′) (Caporaso *et al.*, 2011). MiSeq Illumina DNA sequencing was conducted by Bioengineering Lab using MiSeq Reagent Kit v3 (https://gikenbio.com/).

### Quantitative PCR

The putative benzene degrader *Deltaproteobacterium* Hasda-A, total bacteria, and total archaea in the above biomass samples and samples collected from subsequently transferred cultures were quantified by qPCR using LightCycler 480 II (Roche) (See the detailed protocol in Text S2).

### Sequencing data processing and analysis

Paired-end reads were assembled using PEAR (v 0.9.11) (Zhang *et al.*, 2014). Primers were trimmed with Seqtk (https://github.com/lh3/seqtk). Sequence data were then processed following the UPARSE pipeline (USEARCH v 11.0.667) (Edgar, 2010). Briefly, sequences were strictly filtered (using fastq_filter function) by truncating the sequence length to 250 bp and discarding any sequence if expected errors >0.5. High-quality sequences were dereplicated to obtain unique representative sequences. Chimeras and singleton sequences were discarded before clustering into operational taxonomic units (OTUs) at a default threshold of 97%. The OTU table and representative sequences were imported into QIIME 2 (v 2019.10) (Bolyen *et al.*, 2019) for a downstream analysis. Taxonomic classification was conducted using a Naïve Bayes classifier trained on the 515F/806R primer-specific V4 regions of 16S rRNA gene sequences extracted from Greengenes 13_8 99% OTUs (McDonald *et al.*, 2012). To investigate the shift in the microbial structure of new synthetic medium-based enrichment cultures within 70‍ ‍d of incubation, quantitative indexes (Bray-Curtis dissimilarity and weighted UniFrac) were employed to consider the abundance of microbial phylotypes.

### Statistical analysis

Syntrophic relationships in benzene mineralization under methanogenic conditions were examined using longitudinal samples accumulated in our laboratory (Table S3). Samples included the following: five sediments collected from different places in 2007 together with five corresponding enrichment cultures of these sediments sampled in 2011 (Noguchi *et al.*, 2016), five subcultures from different trials of cultivation methods collected in 2017 (Table S4), and eight samples in the present study. The dataset for samples from the present study consisted of five biological duplicates that were collapsed into average values, and the remaining three were singlet samples. Twenty-three samples were subjected to analyses. Variations in microbial communities between these samples were assessed using a qualitative Jaccard index focusing on the presence/absence of microbial phylotypes at the species level.

To produce correlation matrixes, the OTU table was collapsed at the species level (level 7). Species were filtered out if they had average read counts <2 reads per sample (total count <46 reads) and if they were present in <30% of samples (≤6 samples). All these steps were performed in QIIME 2 (v 2019.10) (Bolyen *et al.*, 2019). Table data were exported to calculate the SparCC correlation matrix. SparCC algorithms (https://bitbucket.org/yonatanf/sparcc) were implemented in Python. The table was also converted into relative abundance to calculate Spearman’s correlation matrix in R (https://www.r-project.org/). Both correlation matrices were filtered to select only species that strongly correlated (correlation coefficient r>0.79 with *P*-value <0.0001) with putative benzene-degrading *Deltaproteobacterium* Hasda-A. The correlation matrix of the selected species including Hasda-A was plotted using the Corrplot package in R. All pairwise plots with a linear Pearson’s regression were prepared using the ggplot2 package in R.

### Chemical analysis

Benzene and methane concentrations were measured by gas chromatography (GC-2010; Shimadzu) equipped with a flame ionization detector and capillary column. The operational protocol was previously described in detail (Masumoto *et al.*, 2012b). Briefly, the temperature was set to 80, 180, and 220°C for the column, injector, and detector, respectively. Helium or nitrogen was used as the carrier gas at a flow rate of 30‍ ‍mL min^–1^. Gas samples (100‍ ‍μL) were collected from the headspace of the culture vials using gastight syringes. The concentrations of benzene and methane were calculated using standard curves. The concentration of benzene in the liquid phase was calculated from the gas phase following Henry’s constant of benzene: *K*_H’_=C_g_/C_l_, where *K*_H’_ is the non-dimensional Henry’s constant for benzene, and C_g_ and C_l_ are the gas phase benzene concentration and liquid phase benzene concentration, respectively, in the same unit (Peng and Wan, 1997). The detection limit of benzene was 0.009‍ ‍mg L^–1^ in the liquid phase.

### Data deposition

Raw sequencing reads from the present study were deposited to the DNA Data Bank of Japan (DDBJ) Sequence Read Archive (DRA) under accession number DRA010466 (Biosample SAMD00235308–SAMD00235325; Bioproject PRJDB10156).

## Results and Discussion

### Elucidation of synthetic medium composition

To achieve the optimal growth of the benzene degrader, potential factors that may affect culture growth were tested. These factors included MgCl_2_, NaCl, riboflavin and prenol, and organic acids. The other components in the medium were set as default concentrations (Table S1).

The concentration of the MgCl_2_.6H_2_O component varied between screened anoxic media, as described in the Materials and Methods section. To optimize the concentration of this component, its concentration was fine-tuned to 0.2 (default), 0.5, 1.0, 1.5, and 3‍ ‍g L^–1^. These cultures were hereafter designated as MgCl_2_ cultures with corresponding concentrations (*e.g.* MgCl_2_ (0.5‍ ‍g L^–1^) and MgCl_2_ (1.0‍ ‍g L^–1^)). Degradation activity was the highest at 0.5‍ ‍g L^–1^ MgCl_2_ (3.6±0.1‍ ‍mg L^–1^ d^–1^), while it was only slightly lower at 0.2 (3.2±0.1‍ ‍mg L^–1^ d^–1^) and 1.0‍ ‍g L^–1^ MgCl_2_ (3.3±0.1‍ ‍mg L^–1^ d^–1^) (Fig. S1). In contrast, higher MgCl_2_ concentrations appeared to induce a negative effect on the degradation activity of cultures, particularly at 3‍ ‍g L^–1^ MgCl_2_ (1.8±0.1‍ ‍mg L^–1^ d^–1^) (Fig. S1).

A salt component (NaCl) is present in some anoxic media of bacterial isolates phylogenetically related to the putative benzene degrader. Therefore, NaCl was added to synthetic medium-based cultures at concentrations of 0.1, 0.5, 1.0, and 5‍ ‍g L^–1^ to clarify whether it promotes benzene degradation activity. These cultures were designated as NaCl cultures with the corresponding added concentrations (*e.g.* NaCl (0.1‍ ‍g L^–1^) and NaCl (0.5‍ ‍g L^–1^)). The addition of NaCl did not exert a clear positive effect on degradation activity. Degradation activity was almost equally high between the default (no NaCl) and 0.1, 0.5, and 1.0‍ ‍g L^–1^ NaCl after day 50 (Fig. S2). There was an accidental dose of a high benzene concentration on day 23 to NaCl (5‍ ‍g L^–1^)-1 (101‍ ‍mg‍ ‍L^–1^) and -2 (268‍ ‍mg‍ ‍L^–1^). While the high concentration were quickly flushed out using the gas mix (80% N_2_: 20% CO_2_ [v/v]) and adjusted to the targeted benzene concentration within 1‍ ‍d, this overdose may have affected benzene degradation. Benzene degradation was slowed in NaCl (5‍ ‍g L^–1^)-1 and stopped in NaCl (5‍ ‍g L^–1^)-2 (Fig. S2).

Previous studies reported carboxylation as a potential initial reaction of anaerobic benzene degradation under nitrate-, iron-, and sulfate-reducing conditions (Abu Laban *et al.*, 2010; Holmes *et al.*, 2011; Luo *et al.*, 2014). Therefore, riboflavin concentrations were increased 100-fold (0.05‍ ‍mg L^–1^ in default media to 5‍ ‍mg L^–1^) and the addition of prenol (a precursor of carboxylase cofactors) (Wang *et al.*, 2018) was examined to establish whether they stimulate benzene degradation activity under methanogenic conditions. The presence of riboflavin at 5‍ ‍mg L^–1^ without (hereafter, riboflavin culture) and with the presence of prenol (5‍ ‍mg L^–1^; hereafter, rib/prenol culture) significantly inhibited benzene degradation activity (Fig. S3). Riboflavin is the precursor of the enzyme cofactors flavin mononucleotide and flavin adenine dinucleotide. Flavin-containing enzymes catalyze common redox reactions and many important processes in cellular metabolism (Wang *et al.*, 2018). Therefore, it remains unclear why the elevated concentration of riboflavin inhibited benzene degradation activity.

A syntrophic relationship may exist for benzene mineralization under methanogenic conditions. The addition of an organic acid as a co-substrate may promote this syntrophic growth. Based on screened media, some organic acids (stearate, butyrate, pyruvate, and gentisic acid) were selected to test their effects on benzene degradation activity (Table S2). The addition of these organic acids (referred to hereafter as the stearate culture, butyrate culture, pyruvate culture, and gentisic acid culture) did not have a clear impact on benzene degradation activity (Fig. S4). Similarly, the addition of yeast extract (0.1‍ ‍g L^–1^) (a complex nutrient source) did not enhance benzene degradation activity (Fig. S4).

**Fig. 1.**
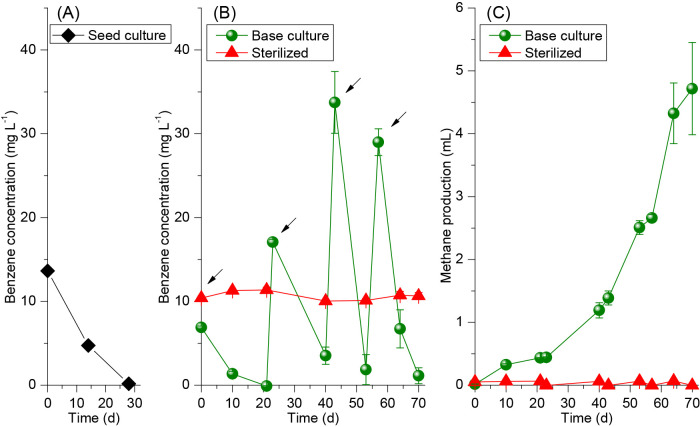
(A): Benzene degradation in the last batch of the seed culture before it was diluted three-fold with the new synthetic medium to set up new enrichment cultures. (B) and (C): Benzene concentrations and methane production, respectively, in synthetic medium-based enrichment cultures (named “Base culture”). Sterilized cultures were used as negative controls. Error bars represent the standard deviation of duplicate samples. Black arrows indicate the times of benzene addition.

Since these results revealed that the default medium was in the range of the optimum composition for the factors tested, this composition was selected for further studies. Benzene degradation was observed without a lag phase after diluting the seed culture with the default medium. Degradation activity was maintained at the same level (~0.5‍ ‍mg‍ ‍L^–1^‍ ‍d^–1^) as that in the seed culture after the dilution (Fig. 1A), and gradually increased to >3.0‍ ‍mg L^–1^ d^–1^ in new synthetic medium-based cultures (referred to hereafter as “base culture”) within 70‍ ‍d of incubation (Fig. 1B). This degradation rate was significantly higher than previously reported values (up to 0.8‍ ‍mg L^–1^ d^–1^) (Masumoto *et al.*, 2014; Noguchi *et al.*, 2016). Consistently, the accumulation of produced methane gradually increased in the vials (Fig. 1C). The ratio of methane produced to benzene degraded was ~4.0, similar to the previously reported values by our colleagues (Sakai *et al.*, 2009) and another research group (Ulrich and Edwards, 2003). This value was slightly higher than the theoretical stoichiometric value of 3.6 (Ulrich and Edwards, 2003), and this difference may be attributed to organic carbon in the remaining soil particles and organic compounds from the detritus cell biomass in the culture. Neither the loss of benzene nor the production of methane was observed in sterilized (negative control) cultures (Fig. 1B and C, “Sterilized”).

### Enrichment of microbial communities

To establish whether our new synthetic medium promoted the growth of benzene-degrading microorganisms, a microbial community analysis was conducted. Twelve cultures were selected to represent three groups: (1) successful enrichment cultures with high benzene degradation activity (>3‍ ‍mg L^–1^ d^–1^) including biological duplicates of the base culture, MgCl_2_ (0.5‍ ‍g‍ ‍L^–1^), and stearate cultures; (2) a sample exhibiting medium activity of 1.5‍ ‍mg L^–1^ d^–1^, for which only one sample was available (NaCl (5‍ ‍g L^–1^) -1); and (3) inhibited samples with no degradation activity, consisting of biological duplicates of riboflavin and rib/prenol cultures, and the NaCl (5‍ ‍g L^–1^) -2 culture. The seed culture was also included as a reference.

**Fig. 2.**
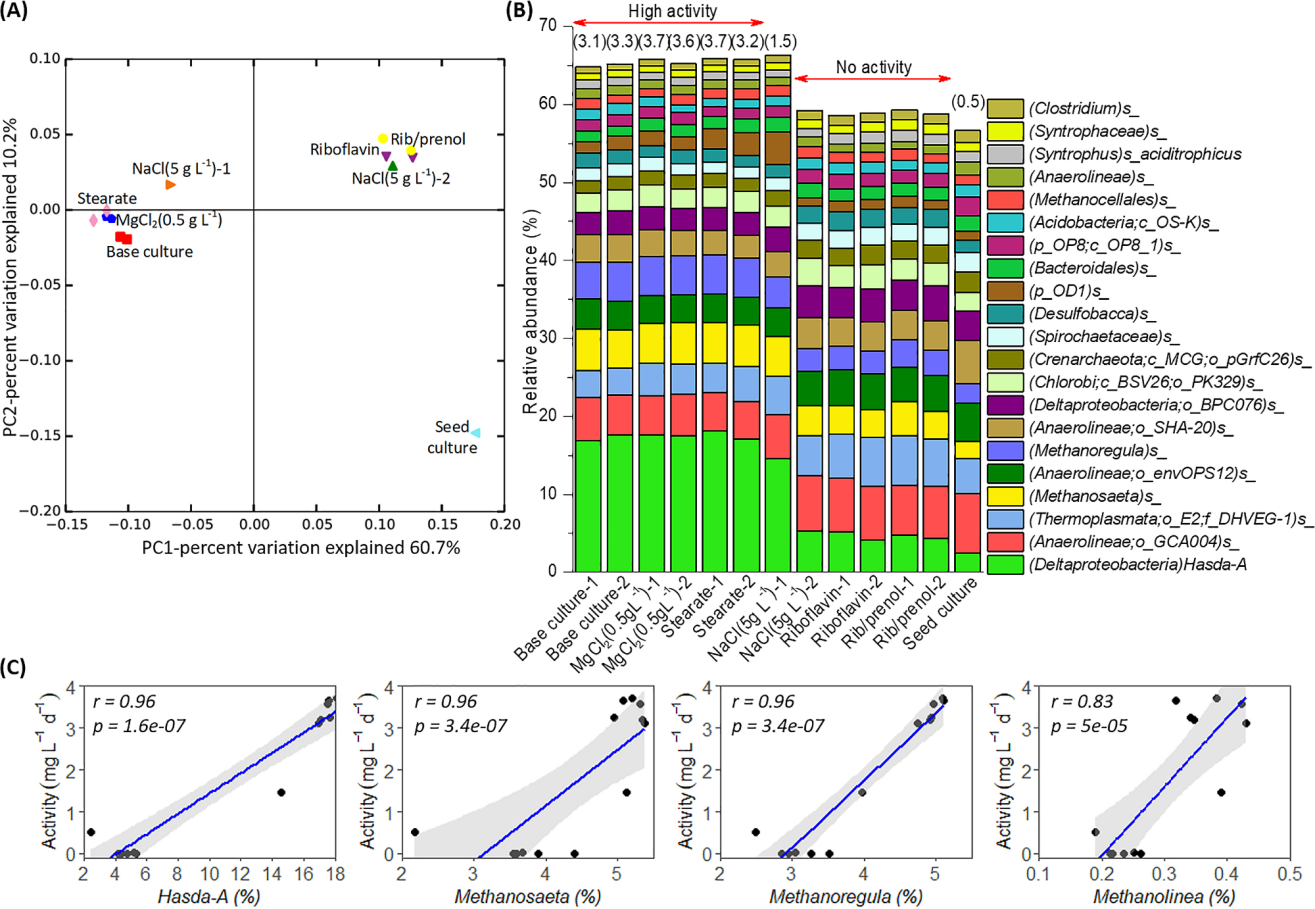
(A) Principal coordinate analysis (PCoA) based on Bray-Curtis dissimilarity of the microbial community between samples. Base culture-1 and -2 (red squares); MgCl_2_ (0.5‍ ‍g L^–1^)-1 and -2 (blue pentagons); stearate-1 and -2 (light pink diamonds); NaCl (5‍ ‍g L^–1^)-1 (orange right-pointing triangle); NaCl (5‍ ‍g L^-1^)-2 (green up-pointing triangle); riboflavin-1 and -2 (purple down-pointing triangles); rib/prenol-1 and -2 (yellow circles); and the seed culture (sky blue left-pointing triangle). (B) The predominant members of microbial communities (average abundance in all samples >1%). Microbial populations are annotated at the lowest level of the known taxonomic classification. “*p_*”, “*c_*”, “*o_*”, “*f_*”, and “*s_*” indicate taxonomic ranks at the phylum, class, order, family, and species level, respectively. The symbol “*s_*” without a taxonomic name at the end indicates the lack of species-level annotation in the reference database. Values in parentheses at the top of each sample indicate benzene degradation activity (mg L^–1^ d^–1^) at the time of sampling. (C) The correlation between putative benzene-degrading *Deltaproteobacterium* Hasda-A and three methanogenic groups (*Methanosaeta*, *Methanoregula*, and *Methanolinea*) at the species level with benzene degradation activity (mg L^–1^ d^–1^). Pearson’s correlation coefficient (r) and significance (*P*-values) are included in each graph. The gray area in each graph represents the 95% confidence interval for predictions from a linear Pearson’s regression.

Differences in the overall microbial composition of samples were assessed using Bray-Curtis dissimilarity. This quantitative index (taking abundance into account) was employed to show the change in microbial communities related to nutrient availability in the synthetic medium within 70‍ ‍d of incubation. A principal coordinate analysis (PCoA) showed the clustering of synthetic medium-based enrichment cultures into two groups according to their degradation activities (Fig. 2A). One cluster contained high-activity samples, including the base culture, MgCl_2_ (0.5‍ ‍g‍ ‍L^–1^), and stearate. The medium-activity sample NaCl (5‍ ‍g L^–1^) -1 also plotted close to this group. The other cluster included the inhibited samples riboflavin, rib/prenol, and NaCl (5‍ ‍g L^–1^) -2. Both groups were distant from the seed culture. This result indicated a significant change in the microbial composition when incubating in the new synthetic medium, even though the incubation time was short (Fig. 2A). A consistent result was obtained when using a weighted UniFrac distance matrix (Fig. S5).

In consistent with PCoA, the taxonomic breakdown at the species level revealed a shift in the relative abundance of the predominant populations (average abundance >1% in all samples) of the microbial community. The most abundant member of the population was putative benzene-degrading *Deltaproteobacterium* Hasda-A (Fig. 2B). Within 70‍ ‍d, the new synthetic medium promoted the growth of Hasda-A from an abundance of 2.45% in the seed culture to >17% in high-activity samples. The relative abundance of Hasda-A was also significantly higher in high-activity samples than in inhibited samples (Fig. 2B). This observation was further supported by the copy number of Hasda-A measured by real-time qPCR (Fig. S6). When translating the relative abundance of Hasda-A into total abundance by multiplying with total bacteria in the sample (assessed by qPCR), the estimated absolute abundance of Hasda-A correlated (Pearson’s correlation of 0.98) with the absolute abundance measured by qPCR (Fig. S7). Estimated values from sequencing were slightly higher than measured values by qPCR. This may be explained by the 16S rRNA gene copy number in bacterial genomes. Our previous work on single-cell genomic and metagenomic data identified only one 16S rRNA gene copy in the Hasda-A draft genome (unpublished data), whereas the total number of bacteria measured by qPCR may be inflated because many bacterial taxa harbor multiple copies of their 16S rRNA gene. Based on the values of the Hasda-A copy number in high-activity samples and the seed culture, the estimated doubling time of Hasda-A in high-activity cultures was approximately 8.2‍ ‍d (Table S5), which was markedly shorter than the average value of approximately 30‍ ‍d previously reported (Ulrich and Edwards, 2003; Luo *et al.*, 2016). In contrast, the doubling time of total bacteria in these samples was 21.1‍ ‍d (Table S5). These results indicated that the selected medium strongly promoted the growth of and enriched Hasda-A.

Among the dominant populations at the species level, similar results to those for Hasda-A were observed for two methanogenic species (belonging to *Methanosaeta* and *Methanoregula*) and one bacterial species belonging to the candidate superphylum OD1 (known as *Ca.* Parcubacteria) (Fig. 2B). The relative abundance of *Methanosaeta* species increased from 2.17% in the seed culture to 5.21±0.17% (*n*=6) in high-activity samples, which was higher than its abundance in inhibited samples (3.82±0.35%, *n*=5). Similarly, the relative abundance of *Methanoregula* species was 4.96±0.13% (*n*=6) in high-activity samples, 3.12±0.27% (*n*=5) in inhibited samples, and 2.49% in the seed culture. The presence of both acetoclastic and hydrogenotrophic methanogens in methanogenic benzene-degrading enrichment cultures was previously reported (Sakai *et al.*, 2009). Another study by our colleagues revealed the incorporation of ^13^C into *Methanosaeta* together with Hasda-A in methanogenic enrichment cultures after an incubation with ^13^C-benzene (Noguchi *et al.*, 2014).

The OD1 species was less abundant than Hasda-A and the two methanogenic populations (*Methanosaeta* and *Methanoregula*). Its average abundance in high-activity samples was 2.07±0.58% (*n*=6), which was almost double of its abundance in the seed culture (1.08%) and inhibited samples (1.25±0.18%, *n*=5). The highest abundance (4.27%) of OD1 species was observed in culture NaCl (5‍ ‍g L^–1^) -1, which exhibited medium benzene degradation activity. A higher death/decay rate in this culture may have released more organic metabolites into the environment, which was beneficial for symbiont microorganisms, such as OD1 bacteria. The abundance of OD1 species in NaCl (5‍ ‍g L^–1^) -2, which lost benzene degradation activity, was only 1.04%. This result indicates the benefits of benzene degradation for the growth of OD1 species. Further discussions of the lifestyle of OD1 bacteria will be presented later in a syntrophic relationship. The OD1 16S rRNA gene sequence V4 region (250 bp) in the present study shares only 94% similarity with the clone OD1 16S rRNA gene (1,435 bp) from the methanogenic benzene-degrading consortium obtained by a research group in Canada (Accession number: *KT025832.1*) (Luo *et al.*, 2016). This research group demonstrated the consistent presence of the OD1 population in methanogenic benzene-degrading enrichment cultures using qPCR (Luo *et al.*, 2016).

The abundance of other dominant populations was not increased in samples exhibiting high benzene degradation activity (Fig. 2B). An important observation was the presence of syntrophic bacteria (*Syntrophus aciditrophicus*, *Desulfobacca*, and unclassified *Syntrophaceae*). Syntrophic bacteria are capable of degrading fatty and aromatic acids, including benzoyl-CoA (a central intermediate of anaerobic benzene degradation), in association with symbiotic partners (*e.g.* methanogens) (McInerney *et al.*, 2007). The occurrence and function of syntrophic bacteria in the methanogenic hydrocarbon-degrading consortia have attracted the attention of researchers, but remain unclear. *Syntrophaceae* populations may consume intermediate products to create thermodynamically favorable conditions for the methanogenic biodegradation of hydrocarbon (McInerney *et al.*, 2007; Toth, 2017). However, they may not be an essential member of the methanogenic benzene-degrading consortia. Regarding methanogenic archaea, *Methanocellales* were consistently observed in these benzene-degrading consortia, indicating a potential role in methanogenesis. The diversity of methanogens may be an essential factor for activity and resilience of these cultures. The remaining populations belonged to diverse microbial groups (*e.g.* *Anaerolineae*, *Bacteroidia*, *Chlorobi*, and *Spirochaetes*). Although these bacteria are often reported for methanogenic hydrocarbon-derived communities (Strąpoć *et al.*, 2011; Toth, 2017), their functions remain elusive.

Based on the shift in microbial populations between high-activity benzene-degrading samples and inhibited samples in the present study, a correlation analysis was conducted to verify microbial populations that positively correlated with benzene degradation activity. The correlation analysis showed that Hasda-A, two hydrogenotrophic methanogens (*Methanoregula* and *Methanolinea*), and one acetoclastic methanogen (*Methanoseta*) strongly correlated with benzene degradation activity (Fig. 2C). The correlation between OD1 species and benzene degradation activity was not strong; as described above, the highest abundance of OD1 species was in the culture NaCl (5‍ ‍g L^–1^) -1. The correlation disappeared with the inclusion of this sample. However, removing this sample from the analysis led to a correlation magnitude of 0.73 (*P*-value=0.007) between the abundance of OD1 species and benzene degradation activity. This result is consistent with previous findings showing a relationship between benzene consumption and the abundance of ORM2 (99% identical to Hasda-A), OD1 bacteria, and *Methanoregula* in methanogenic benzene-degrading enrichment cultures (Luo *et al.*, 2016). In the present study, new synthetic medium-based enrichment cultures mainly promoted the growth of *Deltaproteobacterium* Hasda-A, hydrogenotrophic methanogens (*Methanoregula* and *Methanolinea*), aceticlastic methanogen (*Methanosaeta*), and OD1 bacteria. This result not only supports the previous hypothesis of a syntrophic relationship in the mineralization of benzene, but also demonstrates the greater diversity of the methanogens and methanogenesis pathways involved under methanogenic conditions.

### Towards producing enrichment cultures for bioremediation

**Fig. 3.**
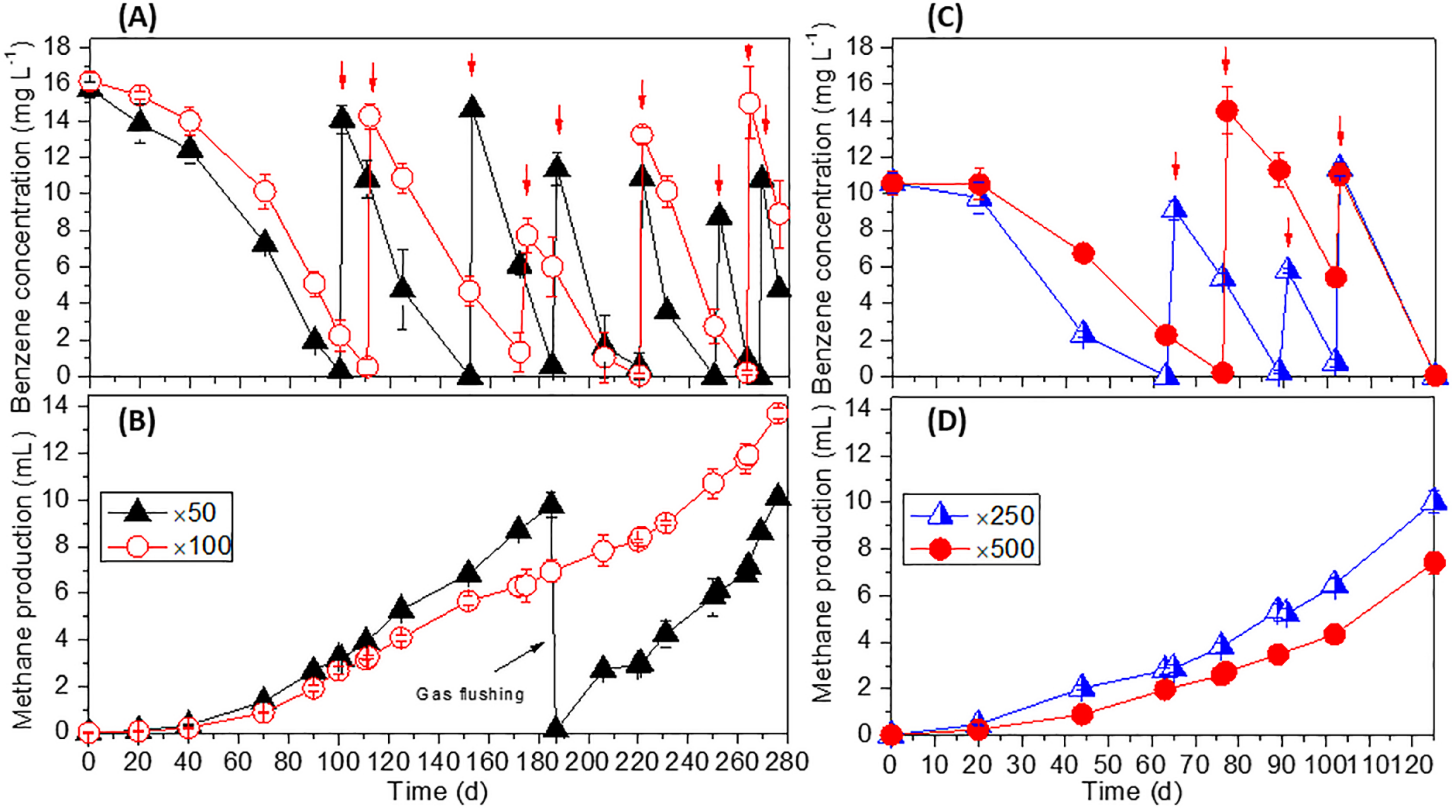
Benzene degradation and methane production in ×50 and ×100 subcultures (A and B) and ×250 and ×500 subcultures (C and D). Error bars represent the standard deviation from triplicate samples (duplicate sample after day 185 for ×50 cultures). Red arrows indicate the time of benzene addition.

One of the current challenges for benzene bioremediation, more specifically bioaugmentation, is the production of a benzene-degrading culture with a high cell density and high degradation activity that facilitates timely and successful remediation. This section investigates whether the synthetic medium designed in the present study may be used to produce this type of capable enrichment culture, namely, if the medium sustains the high degradation activity and growth of benzene-degrading microorganisms in serial dilutions of cultures within a reasonable timeframe. Enrichment cultures exhibiting high activity, as discussed earlier, were employed as “×1” cultures. At the dilution factors 5-times (×5) and 10-times (×10), a negligible decrease was observed in the benzene degradation activity of the enrichment cultures (activity was still >2‍ ‍mg L^–1^ d^–1^ for both dilution factors). Therefore, these cultures were diluted further (×10) into 50-times (×50) and 100-times (×100) subcultures. Benzene degradation was observed without a lag phase; however, degradation activity decreased to 0.09±0.03 and 0.04±0.03‍ ‍mg L^–1^ d^–1^ for subcultures ×50 and ×100, respectively. Importantly, within 125‍ ‍d, the degradation activity of these subcultures increased to 0.47±0.09 and 0.26±0.03‍ ‍mg‍ ‍L^–1^‍ ‍d^–1^, respectively (Fig. 3A). The activity of these subcultures reached nearly 0.9 and 0.51‍ ‍mg L^–1^ d^–1^, respectively, by 275‍ ‍d of incubation. These activities are considered to be higher than those of benzene-degrading methanogenic enrichment cultures reported in previous studies (Masumoto *et al.*, 2014; Luo *et al.*, 2016). The ratios of methane produced (Fig. 3B) to benzene degraded in ×50 cultures were 2.9 and 3.6 for the periods of days 0–185 and 185–275, respectively. This ratio was 3.1 for ×100 cultures throughout the incubation. These ratios were less than that for the ×1 culture and close to or slightly less than the theoretical stoichiometric value, possibly because of a much smaller carbon source other than benzene in the diluted cultures and some remaining organic matter that was not completely mineralized.

The quantification of 16S rRNA gene copy numbers in biomass samples collected from ×50 cultures on day 185 confirmed the significant growth of the putative benzene degrader Hasda-A (Fig. S6). The results obtained showed that the medium mainly sustained the growth of Hasda-A; the abundance of Hasda-A in this subculture was 38% of total bacteria.

On day 185, the ×50 culture (showing activity of 0.48‍ ‍mg‍ ‍L^–1^‍ ‍d^–1^) was diluted 5 and 10 times into 250-times (×250) and 500-times (×500) diluted subcultures. After 20‍ ‍d of incubation, benzene degradation was not observed in ×500 diluted cultures, while degradation activity of 0.04±0.01‍ ‍mg L^–1^ d^–1^ (*n*=3) was recorded for ×250 diluted cultures. The degradation activities of the ×250 and ×500 diluted cultures increased to 0.31±0.03 and 0.16±0.02‍ ‍mg‍ ‍L^–1^‍ ‍d^–1^, respectively, by day 44 (Fig. 3C). Critically, both diluted cultures recovered the degradation activity of their inoculum (0.48‍ ‍mg L^–1^ d^–1^ for ×50 culture on day 185) within 125‍ ‍d of incubation; ×250 cultures achieved degradation activity of 0.49±0.02‍ ‍mg L^–1^ d^–1^, while ×500 cultures exhibited activity of 0.48±0.03‍ ‍mg L^–1^ d^–1^ (Fig. 3C). Methane production was observed accordingly (Fig. 3D). The quick recovery and fast increases in the benzene degradation activity of these subcultures demonstrated the optimal growth of benzene-degrading microorganisms in this defined medium. It is noteworthy to highlight our approach to obtaining this defined formula for the anoxic medium was based on phylogenetic closeness, the expected ecological function, and experimental information. Phylogenetic and ecological closeness was previously demonstrated to be good heuristics for successfully obtaining growth medium (Oberhardt *et al.*, 2015). Despite extensive efforts, there is currently no available medium for the optimal growth of methanogenic benzene-degrading microorganisms. To the best of our knowledge, there is only another research group that has actively and successfully maintained methanogenic benzene-degrading enrichment cultures. Our defined medium is distinct from their medium (Luo *et al.*, 2016). We acknowledge that there are multiple modifications in medium, and further detailed studies are needed to clearly demonstrate the effect of each modification. However, the positive effects of some modifications may be drawn from previous studies. For example, we removed L-cysteine and employed Na_2_S·9H_2_O (0.3‍ ‍g L^–1^) as the sole reducing agent. This may have shortened the lag phase in benzene degradation because L-cysteine was previously demonstrated to be used by microorganisms as an alternative carbon and energy source in methanogenic aromatic hydrocarbon-degrading consortia (Edwards and Grbić-Galić, 1994; Toth, 2017). This effect may be more profound because benzene is a toxic substrate. Our colleague demonstrated that methanogenic benzene-degrading cultures containing L-cysteine as a reductant had higher methane production levels than cultures with only Na_2_S·9H_2_O (unpublished data). A similar effect may be attributed to a reduction in the concentration of nitrilotriacetic acid in a trace element mixture (10-fold lower than the mixture used for a *Syntrophus* bacterium) (Toth, 2017). In addition, a high copy number of the putative benzene degrader Hasda-A in enrichment cultures may be an important factor for the quick recovery of activity. In this case, ×50 diluted cultures had a Hasda-A 16S rRNA gene copy number of 1.8×10^7^ copies mL^–1^ on day 185 (Fig. S6), which indicates that the ×250 and ×500 subcultures had approximately 3.7×10^6^ and 1.8×10^6^ copies mL^–1^, respectively, on day 0. These copy numbers of Hasda-A may be a suitable cell density for their communication to induce degradation activity. Cell density-dependent activity was previously reported for anaerobic slow-growing bacteria (Oshiki *et al.*, 2020).

We would also like to emphasize that the putative benzene degrader, Hasda-A, is far from the closest isolate (it shares less than 90% identity in the 16S rRNA gene sequence). Moreover, media for other aromatic compound degraders may not necessarily be suitable for benzene degraders. Although the default medium was obtained based on limited information, it improved benzene degradation and was the best among the various conditions tested. The compositions of this medium provided appropriate nutritional requirements to promote the growth of benzene degraders under methanogenic conditions. In short, the experimental results obtained demonstrated the feasibility of producing high-capability enrichment cultures for the effective bioremediation of benzene using this defined medium.

Bioremediation is a promising approach for the removal of benzene to the very low levels required by environmental standards (0.01 [Japan] and 0.005 [US] mg L^–1^); this is challenging for other methods (*i.e.*, physicochemical treatments). Bioremediation is the best choice for contamination at sub-mg L^–1^ to 10‍ ‍mg L^–1^ levels, while physicochemical treatments have advantages at higher benzene concentrations. Effective bioremediation is only possible if bioaugmented cultures maintain degradation activity at these low substrate concentrations. Triplicates of cultures ×50 and ×100 were incubated at low benzene concentrations (1–2‍ ‍mg L^–1^). A long-term incubation demonstrated that these cultures maintained benzene degradation activity over a long period (>320 d) at these low benzene concentrations, and benzene was degraded to below the detection limit (0.009‍ ‍mg L^–1^) (Fig. S8). These cultures may achieve benzene degradation to a concentration that at least meets the environmental standard in Japan.

### Insights into the syntrophic relationship between benzene-degrading microorganisms under methanogenic conditions

Previous studies reported the dominance of the microbial consortium in methanogenic cultures incubated long-term with benzene as the only carbon and energy source (Sakai *et al.*, 2009; Luo *et al.*, 2016), indicating a potential syntrophic relationship in the methanogenic mineralization of benzene. Insights into this coordination may provide a better understanding of the factors affecting the growth and activity of benzene-degrading microorganisms. Therefore, we conducted a correlation analysis employing the longitudinal data of microbial communities collected from our methanogenic benzene-degrading cultures to examine this proposed syntrophic interaction.

**Fig. 4.**
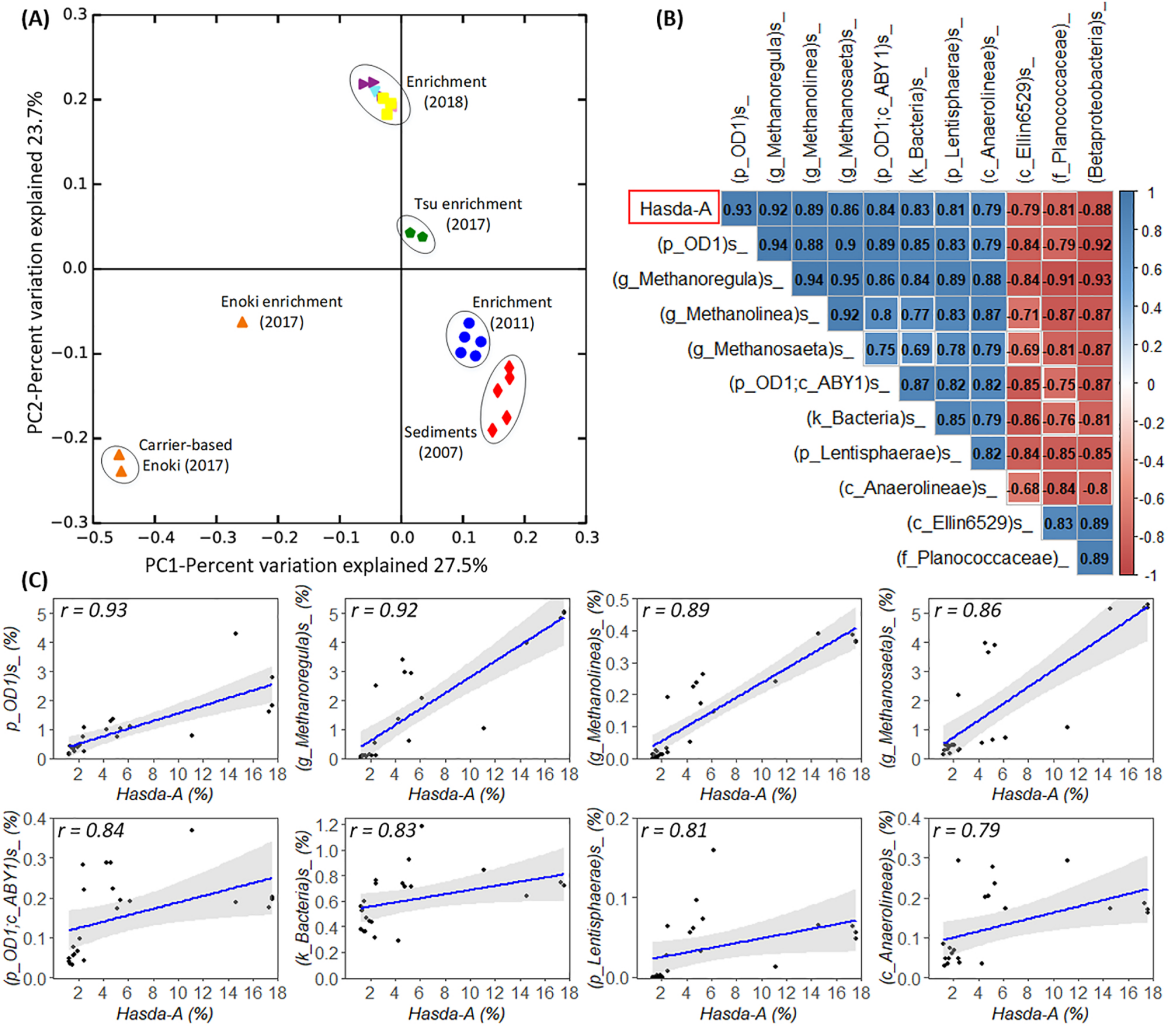
(A) Jaccard-based PCoA of 23 samples calculated at the species level of the microbial community. Numbers in parentheses indicate the years of biomass sampling. “Tsu” and “Enoki” stand for “Tsuchiura” and “Enokibashi”, names according to the geographical sites in Japan from which the original sediments were collected for enrichment. Carrier-based Enoki (2017) are enrichment cultures derived from the Enoki enrichment by selecting only microbes attached to artificial carriers. (B) SparCC correlation matrix of microorganisms having a strong correlation with *Deltaproteobacterium* Hasda-A (correlation coefficients >0.79 and two-sided pseudo *P*-value<0.0001 based on the bootstrapping of 100 repetitions). The value of the pairwise correlation coefficient is shown in each box of the grid. The annotation of the microbial population includes the lowest known taxonomy in parentheses. “*k_*”, “*p_*”, “*c_*”, “*o_*”, “*f_*”, “*g_*”, and “*s_*” indicate taxonomic ranks at the kingdom, phylum, class, order, family, genus, and species level, respectively. The symbol “*s_*” without a taxonomic name at the end indicates the lack of species-level annotation in the reference database. (C) Pairwise plot of relative abundance between Hasda-A and microbial species (from 23 samples) showing strong positive correlations by the SparCC algorithm. The gray area in each graph represents a 95% confidence interval for predictions from a linear Pearson’s regression.

The overall variation of microbial communities between 23 longitudinal samples was shown using PCoA of the Jaccard index (Fig. 4A). Although sediment samples were collected from different places in 2007, 4 years of enrichment in the laboratory under the same culture conditions (diluted in Milli-Q water with benzene as the only carbon source) led to a similar structure in their microbial communities, as demonstrated by the close grouping of the five corresponding enrichments (see the blue circles in Fig. 4A). This result indicates the selection of benzene-degrading microorganisms and supporting microbial communities (Noguchi *et al.*, 2016). In a comparison of enrichment cultures in our laboratory collected in 2011, 2017, and 2018, incubation times and different cultivation techniques markedly changed the microbial composition of the cultures, as demonstrated by the distinct clustering of these cultures (Fig. 4A). All 2018 enrichment cultures in the present study were derived from the same seed culture and used the same synthetic medium with a cultivation period of only 70 d; therefore, the microbial composition (presence/absence) of these samples was very similar, as demonstrated by their tight grouping in Fig. 4A. Samples collected in 2017 were derived from the Tsu and Enoki enrichments (“Tsu” and “Enoki” stand for “Tsuchiura” and “Enokibashi”, respectively, names according to geographical locations in Japan from which the original sediments were collected in 2007 for enrichment) (Noguchi *et al.*, 2016). Inhibited and non-inhibited samples of the Tsu enrichment were experimentally incubated for only 112‍ ‍d (Table S4). Therefore, they were closely clustered together regardless of their different benzene degradation activities. Regarding the Enoki enrichment and its artificial carrier-based cultures, although the experimental time of transfer and incubation was only approximately 165‍ ‍d (Table S4), the two carrier-based cultures solely contained microorganisms that attached on the carriers. As a result, the microbial components of these two cultures significantly differed from the inoculum (the Enoki enrichment). In addition to PCoA, a taxonomic breakdown of the dominant microbial compositions was added to Table S6 in order to demonstrate the shift in microbial communities between samples.

Regardless of variations in microbial communities, the correlation analysis aims to identify the co-occurrence of microorganisms in these samples. Previous studies recommended the SparCC algorithm based on Aitchison’s log-ratio analysis as an appropriate correlation method to deal with compositional data generated by 16S rRNA gene amplicon sequencing (Friedman and Alm, 2012; Weiss *et al.*, 2016). A classical method, Spearman’s correlation, was also used as a complementary analysis (Weiss *et al.*, 2016).

Although differences were observed in the order of the correlation magnitude, SparCC and Spearman’s correlation methods both consistently showed a strong positive correlation between Hasda-A with populations belonging to OD1 species and three methanogenic species (belonging to *Methanoregula*, *Methanolinea*, and *Methanosaeta*) (Fig. 4B and S9). Other than these groups, the two methods indicated different microbial groups having strong correlations with Hasda-A. Additionally, Spearman’s method is more likely to indicate negative correlations than SparCC because of the nature of the compositional data that SparCC was developed to cope with (Friedman and Alm, 2012). The results of the SparCC correlation analysis were presented by relative abundance and a linear Pearson’s correlation that also supported the correlation of Hasda-A with OD1 and methanogens (Fig. 4C).

The positive correlation indicated the potentially syntrophic relationship Hasda-A has with OD1 and methanogens (*Methanoregula*, *Methanolinea*, and *Methanosaeta*) in methanogenic benzene-degrading enrichment cultures. The magnitude of the SparCC correlation coefficient of Hasda-A was the highest with OD1 species (0.93), followed by the hydrogenotrophic methanogens *Methanoregula* (0.92) and *Methanolinea* (0.89), and then the acetoclastic methanogen *Methanosaeta* (0.86). The coordination between these microorganisms may be essential for the mineralization of benzene under methanogenic conditions. This result supported observations of the dominant microbial consortium in methanogenic benzene-degrading cultures. Correlation results were supported by experimental observations, as discussed earlier, demonstrating that these microorganisms were the only microbial groups showing positive correlations with benzene-degrading activity.

Candidate superphylum OD1 (known as *Ca.* Parcubacteria) has been observed in a wide range of anoxic environments without laboratory culture representatives. They have small genomes (<1.5‍ ‍Mb) with reduced metabolic capabilities (Nelson and Stegen, 2015; León-Zayas *et al.*, 2017). OD1 bacteria were previously proposed to have fermentative lifestyles obtaining metabolites/nutrients/energy from partners/host microorganisms via close cell–cell contact using type IV pili. The archetype metabolism of OD1 bacteria is the production of H_2_, acetate, or other fermentative products (Nelson and Stegen, 2015; Castelle *et al.*, 2018). Based on these preliminary observations, OD1 species may utilize intermediate products (*e.g.* fatty acids) from benzene degradation by Hasda-A and then produce acetate/hydrogen for methanogens. Another possible scenario is that OD1 populations are scavengers of metabolized products in cultures, not only from benzene degraders, but also from the microbial biomass. This may explain the highest abundance of OD1 in the benzene-degrading NaCl (5‍ ‍g L^–1^) -1 culture that possibly had a higher decay rate on the microbial biomass. OD1 was not detected in our previous study using stable isotope probing with ^13^C-labeled benzene (Noguchi *et al.*, 2014). This may be attributed to the minimal incubation time used to minimize the cross-feeding of labeled compounds. Further studies are needed to define the role of OD1 species in benzene mineralization.

The presence of hydrogenotrophic and aceticlastic methanogens suggested the production of acetate and hydrogen from anaerobic benzene degradation under methanogenic conditions. The results of the correlation analysis demonstrated, for the first time, the involvement of *Methanolinea*, hydrogenotrophic methanogens, in anaerobic benzene degradation. Although *Methanolinea* were less abundant in cultures than other methanogens, they were consistently present in samples (Fig. 4C). This result showed the diversity and functional redundancy of methanogens in the methanogenic benzene-degrading consortia. This factor may be important for the resilience of our enrichment cultures to disturbances, such as upon dilution into new medium. The involvement of methanogenesis in methanogenic benzene degradation was confirmed in inhibition experiments: the addition of bromoethanesulfonate (final concentration 5‍ ‍mM), an inhibitor of methanogenesis, completely inhibited both benzene degradation and methane production within 6‍ ‍d (Fig. S10).

To obtain a more detailed understanding of the methanogenic benzene-degrading consortia, the correlation network of dominant microbial populations (presented in Fig. 2B) was focused on (Fig. S11). Within this context, Hasda-A and OD1 have similar networks with only additional connections to the *Anaerolineae* and *Syntrophaceae* phylotypes (*Desulfobacca* and unclassified *Syntrophaceae*). In contrast, methanogens positively correlated with many bacterial groups, including functionally uncharacterized bacteria (*Anaerolineae*, *Acidobacteria*, and *Chlorobi*). These uncharacterized bacteria tightly coordinated with each other. Their consistent presence and complex interactions with methanogens indicate their ecological role in the flow of carbon under methanogenic conditions.

In summary, the present study successfully developed a defined formula for anoxic medium for the optimized cultivation of benzene-degrading microorganisms under methanogenic conditions. The medium was demonstrated to mainly sustain the growth of the benzene degrader *Deltaproteobacterium* Hasda-A and coordinating microorganisms. It enhanced degradation activity and shortened the lag phase of subcultures, which are promising for the production of highly capable cultures to elucidate the underlying molecular mechanisms and develop practical applications. The correlation analysis revealed potential syntrophic relationships between Hasda-A, OD1 bacteria, acetoclastic methanogens (*Methanosaeta*), and hydrogenotrophic methanogens (*Methanoregula* and *Methanolinea*) in the methanogenic mineralization of benzene. Inhibition experiments confirmed the involvement of methanogenesis in anaerobic benzene degradation. Further studies are needed to verify the role of OD1 bacteria.

## Citation

Phan, H. V.., Kurisu, F., Kiba, K., and Furumai, H. (2021) Optimized Cultivation and Syntrophic Relationship of Anaerobic Benzene-Degrading Enrichment Cultures under Methanogenic Conditions. *Microbes Environ ***36**: ME21028.

https://doi.org/10.1264/jsme2.ME21028

## Supplementary Material

Supplementary Material
