# Supplementary material for: Optimized Cultivation and Syntrophic Relationship of Anaerobic Benzene-Degrading Enrichment Cultures under Methanogenic Conditions
Source: Microbes Environ. 2021 Aug 24;36(3):ME21028. doi: 10.1264/jsme2.ME21028 (PMC8446749; doi:10.1264/jsme2.ME21028)
Supplement: Supplementary file 1 — Supplementary Material [file 36_21028_s1.pdf]

SUPPORTING INFORMATION

# Optimized cultivation and syntrophic relationship of anaerobic benzene-degrading enrichment cultures under methanogenic conditions

***Microbes and Environments***

Hop V. Phan[1], Futoshi Kurisu[2*], Koichiro Kiba[3] and Hiroaki Furumai[2]

*[1]JSPS International Research Fellow, Research Center for Water Environment Technology, The University of Tokyo, 7–3–1, Hongo, Bunkyo, Tokyo 113–8656, Japan.*

*[2]Research Center for Water Environment Technology, The University of Tokyo, 7–3–1, Hongo, Bunkyo, Tokyo 113–8656, Japan.*

*[3]Department of Urban Engineering, Graduate School of Engineering, The University of Tokyo, 7–3–1, Hongo, Bunkyo, Tokyo 113–8656, Japan.*

[*]Corresponding author: kurisu@env.t.u-tokyo.ac.jp; Ph: +81-3-5841-7445

**Text S1 (**The list of DSMZ media used for screening to select the synthetic medium in this study): *Desulfobacteraceae* media DSMZ 23472, DSMZ 15576, DSMZ 18843, and DSMZ 18734; Toluene degrader DSMZ 383a and DSMZ 612; Phenol and benzoate degraders media DSMZ 14795, DSMZ 26646, and DSMZ 14947, *Syntrophus* media DSMZ 8423 and DSMZ 102352, and *Smithella* medium DSMZ 16934.

**Table S1**: The formulation and preparation of the synthetic medium

| **Synthetic medium** | |
|---|---|
| $KH_2PO_4$ | 0.20 g |
| $NH_4Cl$ | 0.30 g |
| $MgCl_2.6H_2O$ | 0.20 g |
| $CaCl_2.2H_2O$ | 0.15 g |
| $NaHCO_3$ solution | 50 mL |
| $Na_2S.9H_2O$ | 0.30 g |
| Na-resazurin solution (2.5 g $L^{-1}$) | 0.4 mL |
| Trace element solution | 1.00 mL |
| Vitamin mixture solution | 1.00 mL |
| Thiaminine-$HCl.2H_2O$ solution | 1.00 mL |
| Vitamin B12 solution | 0.10 mL |
| Riboflavin solution | 1.00 mL |
| Distilled water | To 1000 mL |
| | |
| **$NaHCO_3$ solution** | |
| $NaHCO_3$ | 2.5 g |
| Distilled water | 50 mL |
| | |
| **Trace element solution** | |
| Nitrilotriacetic acid (NTA) | 1.50 g |
| $FeCl_3.6H_2O$ | 1.35 g |
| $MnCl_2.4H_2O$ | 0.10 g |
| $CoCl_2.6H_2O$ | 24.00 mg |
| $CaCl_2.2H_2O$ | 0.10 g |
| $ZnCl_2$ | 0.10 g |
| $CuCl_2.2H_2O$ | 25.00 mg |
| $Na_2MoO_4.2H_2O$ | 24.00 mg |
| $H_3BO_3$ | 0.01 g |
| $NiCl_2.6H_2O$ | 24.00 mg |
| Distilled water | To 1000 mL |
| | |

| | |
|---|---|
| **Selenite-tungstate solution** | |
| NaOH | 400 mg |
| $Na_2WO_4.2H_2O$ | 3.00 mg |
| $Na_2SeO_3.5H_2O$ | 4.00 mg |
| Distilled water | To 1000 mL |
| | |
| **Vitamin mixture solution** | |
| $NaH_2PO_4$ + $Na_2HPO_4$ (10 mM total P; pH 7.1) | 100 mL |
| Biotin | 2.00 mg |
| Folic acid | 2.00 mg |
| Pyridoxine-HCl | 10.00 mg |
| Nicotinic acid | 5.00 mg |
| D-Ca-pantothenate | 5.00 mg |
| p-Aminobenzoic acid | 5.00 mg |
| Lipoic acid | 5.00 mg |
| | |
| **Thiamine solution** | |
| $H_3PO_4$ + $NaH_2PO_4$ (10 mM total P; pH 3.4) | 100 mL |
| Thiamine-HCl・$2H_2O$ | 5.00 mg |
| | |
| **Riboflavin solution** (heating to ~50°C) | |
| $H_3PO_4$ + $NaH_2PO_4$ (10 mM total P; pH 3.2) | 100 mL |
| Riboflavin | 5.00 mg |
| | |
| **Vitamin B12 solution** | |
| Distilled water | 100 mL |
| Vitamin B12 | 1.00 mg |

**Table S2**: List of the investigated parameters and concentrations (the concentrations were chosen based on previous studies and/or low enough to not serve as alternative carbon sources for microbial growth)

| **Components** | **Amount per 1000 mL** |
|---|---|
| **Organic acids** | |
| Na-stearate | 0.01 g |
| Na-pyruvate | 0.01 g |
| Na-butyrate | 0.01 g |
| Gentisic acid | 0.01 g |
| **Salts and concentrations** | |
| $MgCl_2.6H_2O$ | 0.5; 1.0; 1.5; 3.0 g |
| NaCl | 0.1; 0.5; 1.0; 5.0 g |

| Complex growth component | |
|---|---|
| Yeast extract | 0.1 g |
| **Cofactors of carboxylation** | |
| Riboflavin | 5.0 mg |
| Riboflavin + Prenol | 5.0 mg + 5.0 mg |

Notes: *Each chemical solution was prepared under strictly anoxic condition and then injected into the base medium cultures to reach the final concentration specified in the table.*

**Text S2:** Quantitative PCR

The putative benzene degrader *Deltaproteobacterium* Hasda-A, total bacteria, and total archaea in the above biomass samples and samples collected from subsequently transferred cultures were quantified by qPCR using a LightCycler 480 II (Roche, Switzerland). The SYBR Green I method was employed for quantifying 16S rRNA gene copy numbers of Hasda-A and total archaea using primer pairs 207F (5′-CTCTGTCTCAAGTTGCCGCTTA-3′)/437R (5′-TACGTTTCGTCCCTTCAAACA-3′) (Sakai et al., 2009) and Arch-967F (5′-AATTGGCGGGGGAGCAC-3′)/Arch-1060R (5′-GGCCATGCACCWCCTCTC-3′) (Bengtson et al., 2012), respectively. Total bacteria were determined using the *TaqMan* probe method with primers 331F (5′-TCCTACGGGAGGCAGCAGT-3′)/797R (5′-GGACTACCAGGGTATCTAATCCTGTT-3′) and fluorescent probe (5′-FAM-CGTATTACCGCGGCTGCTGGCAC-TAM-3′) (Kasuga et al., 2010). Detailed PCR conditions have been described previously (Kasuga et al., 2010) with primer-specific annealing temperatures. All qPCR assays were performed in triplicate. Absolute quantification was performed using standard curves generated from 10-fold serial dilution ($10^2$–$10^7$ copies/reaction) of DNA standards. The DNA standard templates were prepared by cloning 16S rRNA gene sequences into pDrive Cloning Vector (PCR Cloning Plus Kit, Qiagen). For Hasda-A, the standard template was a gel-purified plasmid containing 16S rRNA gene fragments of Hasda-A. For total bacteria and

total archaea, DNA standard templates were generated by cloning nearly full-length 16S rRNA gene sequences amplified (using universal bacterial and archaeal primer sets, respectively) from total DNA extract of our enrichment cultures into vector pDrive. Plasmids were gel-purified and the fragment-containing targets were amplified with a plasmid-specific M13 primer set. The purified M13 PCR products were used as standard templates.

**Table S3**: List of samples employed for correlation analysis

| Samples[a] | Activity (mg $L^{-1}$ $d^{-1}$) | Culture medium[b] | Treatment | Collected year | DNA extraction[c] | Sequencing[d] | Amplicon[e] | Sources |
|---|---|---|---|---|---|---|---|---|
| Base culture | 3.2 | New medium | None | 2018 | FastDNA | MiSeq | V4 region | This study |
| $MgCl_2$ (0.5g $L^{-1}$) | 3.61 | New medium | $MgCl_2$ (0.5 g $L^{-1}$) | 2018 | FastDNA | MiSeq | V4 region | This study |
| Stearate | 3.35 | New medium | Stearate | 2018 | FastDNA | MiSeq | V4 region | This study |
| NaCl (5g $L^{-1}$)-1 | 1.45 | New medium | NaCl (5 g $L^{-1}$) | 2018 | FastDNA | MiSeq | V4 region | This study |
| NaCl (5g $L^{-1}$)-2 | 0.00 | New medium | NaCl (5 g $L^{-1}$) | 2018 | FastDNA | MiSeq | V4 region | This study |
| Riboflavin | 0.01 | New medium | Riboflavin | 2018 | FastDNA | MiSeq | V4 region | This study |
| Rib/prenol | 0.00 | New medium | Riboflavin & Prenol | 2018 | FastDNA | MiSeq | V4 region | This study |
| Seed | 0.50 | Sterilized water | None | 2018 | FastDNA | MiSeq | V4 region | This study |
| Tsu-Positive | 2.00 | Sterilized water | None | 2017 | FastDNA | MiSeq | V4 region | This study |
| Tsu-Negative | 0.00 | Sterilized water | Inhibition | 2017 | FastDNA | MiSeq | V4 region | This study |
| Enokibashi | 0.50 | Sterilized water | None | 2017 | FastDNA | MiSeq | V4 region | This study |
| Enokibashi α | 0.30 | Synthetic medium | Artificial carriers | 2017 | FastDNA | MiSeq | V4 region | This study |
| Enokibashi β | 0.28 | Synthetic medium | Artificial carriers | 2017 | FastDNA | MiSeq | V4 region | This study |
| TA-Enrichment | 0.20 | Sterilized water | None | 2011 | ISOIL | 454 FLX | V4 region | (Noguchi et al., 2016) |

| | | | | | | | | |
|---|---|---|---|---|---|---|---|---|
| TB-Enrichment | 0.40 | Sterilized water | None | 2011 | ISOIL | 454 FLX | V4 region | (Noguchi et al., 2016) |
| IW-Enrichment | 0.10 | Sterilized water | None | 2011 | ISOIL | 454 FLX | V4 region | (Noguchi et al., 2016) |
| SN-Enrichment | 0.20 | Sterilized water | None | 2011 | ISOIL | 454 FLX | V4 region | (Noguchi et al., 2016) |
| TE-Enrichment | 0.30 | Sterilized water | None | 2011 | ISOIL | 454 FLX | V4 region | (Noguchi et al., 2016) |
| TA-Sediment | 0.00 | N/A | None | 2007 | ISOIL | 454 FLX | V4 region | (Noguchi et al., 2016) |
| TB-Sediment | 0.00 | N/A | None | 2007 | ISOIL | 454 FLX | V4 region | (Noguchi et al., 2016) |
| IW-Sediment | 0.00 | N/A | None | 2007 | ISOIL | 454 FLX | V4 region | (Noguchi et al., 2016) |
| SN-Sediment | 0.00 | N/A | None | 2007 | ISOIL | 454 FLX | V4 region | (Noguchi et al., 2016) |
| TE-Sediment | 0.00 | N/A | None | 2007 | ISOIL | 454 FLX | V4 region | (Noguchi et al., 2016) |

***(a)*** *See Table S4 for detail experiment of samples collected in 2017.* ***(b)*** *Sterilized water: Wet sediments (0.5-1 g $mL^{-1}$ water) were suspended in sterilized ultrapure water with benzene as only carbon and energy source, and $Na_2S$ (0.3 g $L^{-1}$) & L-cysteine (0.3 g $L^{-1}$) as reducing agents.* ***N/A:*** *not applicable.* ***(c)*** *DNA extraction kits: FastDNA SPIN Kit for Soil and the FastPrep Instrument (MP Biomedicals, Santa Ana, USA) and ISOIL for Beads Beating (Nippon Gene, Tokyo, Japan).* ***(d)*** *Sequencing platforms: MiSeq Illumina platform and Roche 454 FLX Titanium platform.* ***(e)*** *V4 region of the 16S rRNA genes.*

**Table S4**: Detail experiments of cultures collected in 2017 used for correlation analysis

<table>
<tr><th>Symbols</th><th>Cultures</th><th>Description</th></tr>
<tr><td rowspan="2">Tsu, 2017</td><td>Tsuchiura, inhibition</td><td>15 mL of Tsuchiura soil enrichment culture was transferred to 72 mL vial. Ethylene was added to 0.1% headspace concentration. Incubated for 116 d with benzene. Inhibition was observed and initial benzene addition was only slightly reduced. Biomass sample was collected on day 112.</td></tr>
<tr><td>Tsuchiura, non-inhibition</td><td>The remaining 70 mL of the same Tsuchiura culture was continuously fed with benzene. Benzene degradation activity was observed during all the incubation period of 116 d. Biomass sample was collected on day 112. Degradation activity up to 2 mg $L^{-1}$ $d^{-1}$.</td></tr>
<tr><td>Enoki, 2017</td><td>Enokibashi</td><td>Soil enrichment culture that is seed culture for both Enokibashi α and β cultures. Degradation activity ~ 0.5 mg $L^{-1}$ $d^{-1}$.</td></tr>
<tr><td rowspan="2">Carrier-based Enoki, 2017</td><td>Enokibashi α</td><td rowspan="2">To establish a soil-free cultures, artificial carriers were added to soil enrichment culture (Enokibashi) and cultivated for 50 d. Carriers and supernatant (20 mL) were transferred to new bottles and further cultivated for 45 d. After that, the carriers were transferred to a new bottle containing the same synthetic medium, but different in medium concentration: 20 times diluted (Enokibashi α) and 100 times diluted (Enokibashi β) medium concentration. Both cultures were continuously incubated with benzene and biomass samples were collected on day 68. Degradation rate was ~ 0.2 mg $L^{-1}$ $d^{-1}$ for both.</td></tr>
<tr><td>Enokibashi β</td></tr>
</table>

*"Tsuchiura" and "Enokibashi" are the names of two geographical locations in Tokyo, Japan where the original sediments were collected in 2007 for enrichment.*

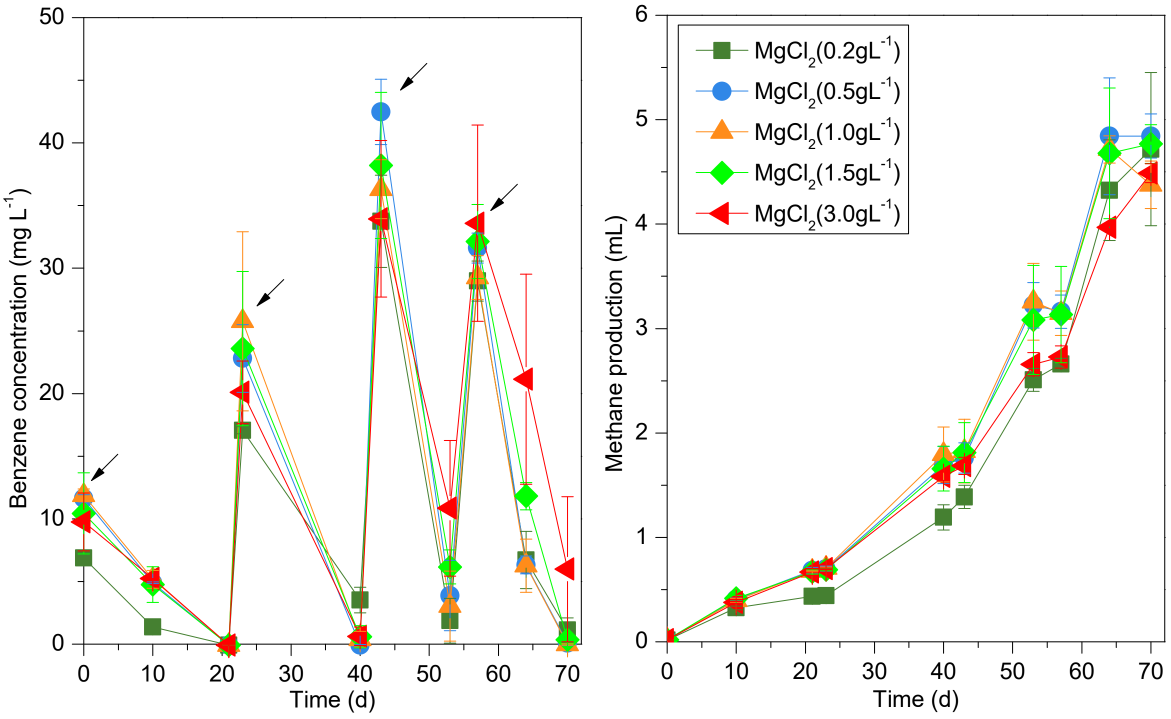


**Figure S1**: Benzene concentrations and methane production in synthetic medium-based cultures with different concentrations of $MgCl_2.6H_2O$ that was fine-tuned to 0.2 (default), 0.5, 1.0, 1.5, and 3.0 g $L^{-1}$. These cultures were designated as $MgCl_2$ cultures accordingly with corresponding

concentrations. Error bars represent the standard deviation of duplicate samples. Black arrows indicate the times of benzene addition.

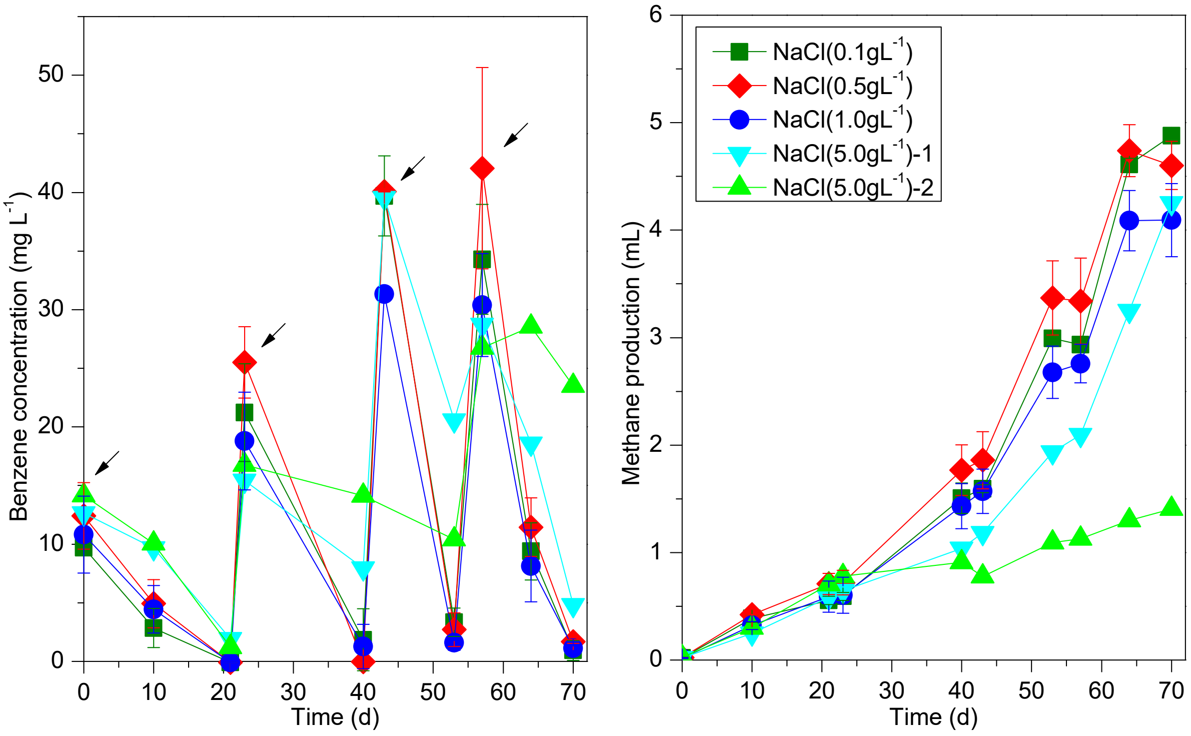


**Figure S2**: Effect of NaCl addition on benzene degradation and methane production in synthetic medium-based enrichment cultures. NaCl was added to the synthetic medium-based cultures at concentration of 0.1, 0.5, 1.0 and 5.0 g $L^{-1}$ that were named accordingly as NaCl cultures with corresponding concentrations. Error bars represent the standard deviation of duplicate samples, except for NaCl(5.0g$L^{-1}$)-1 and -2. On day 23, there was an overdose of benzene to NaCl(5g$L^{-1}$)-1 (101 mg $L^{-1}$) and -2 (268 mg $L^{-1}$). High benzene concentrations were quickly flushed out using gas mix (80% $N_2$ : 20% $CO_2$, v/v) and adjusted to the targeted concentrations within a day.

Degradation activity was decreased in NaCl(5gL$^{-1}$)-1 while it was stopped in NaCl(5gL$^{-1}$)-2. Black arrows indicate the times of benzene addition.

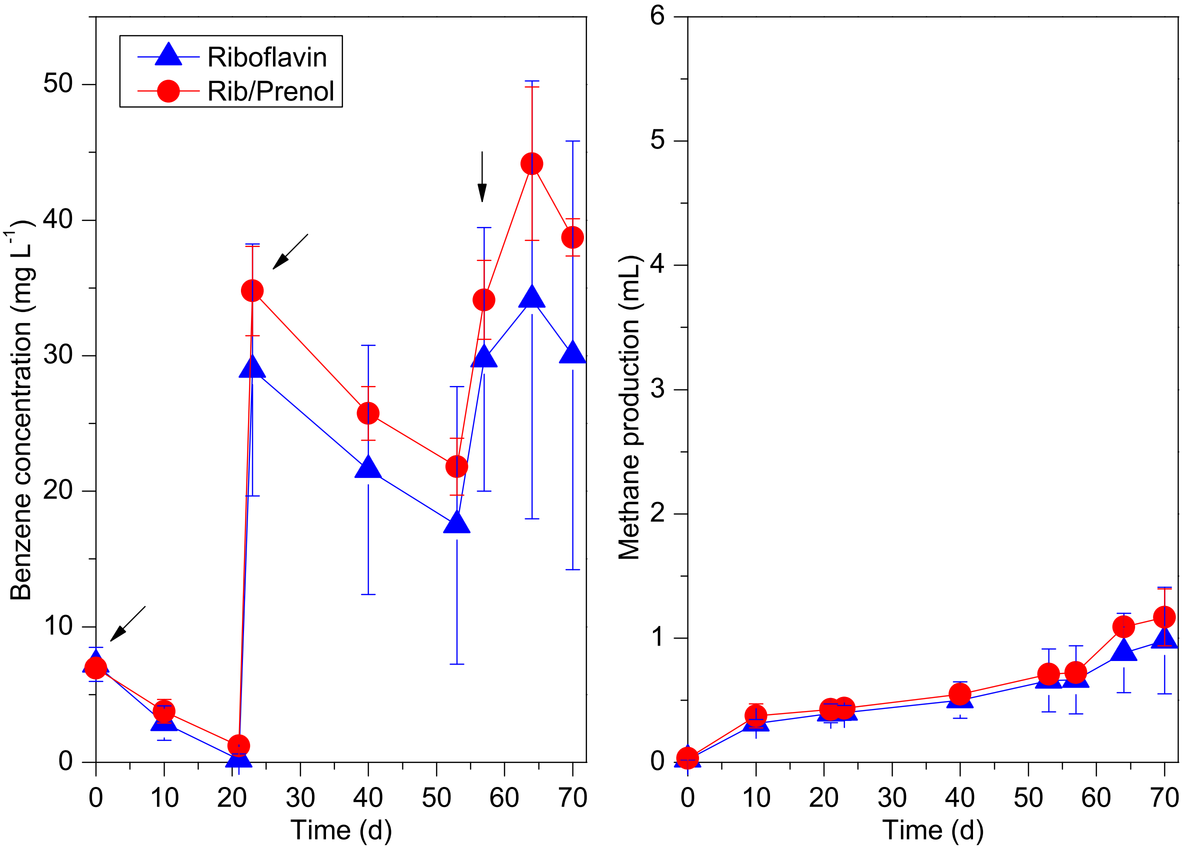


**Figure S3**: Benzene degradation and methane production in synthetic-based enrichment cultures supplied with cofactors of carboxylation. Riboflavin concentration was increased to 5 mg L$^{-1}$ (100 times of default concentration) (namely Riboflavin cultures), and with the addition of Prenol (5 mg L$^{-1}$) (namely Rib/Prenol cultures). Error bars indicate the standard deviation of duplicate cultures. Black arrows indicate the times of benzene addition.

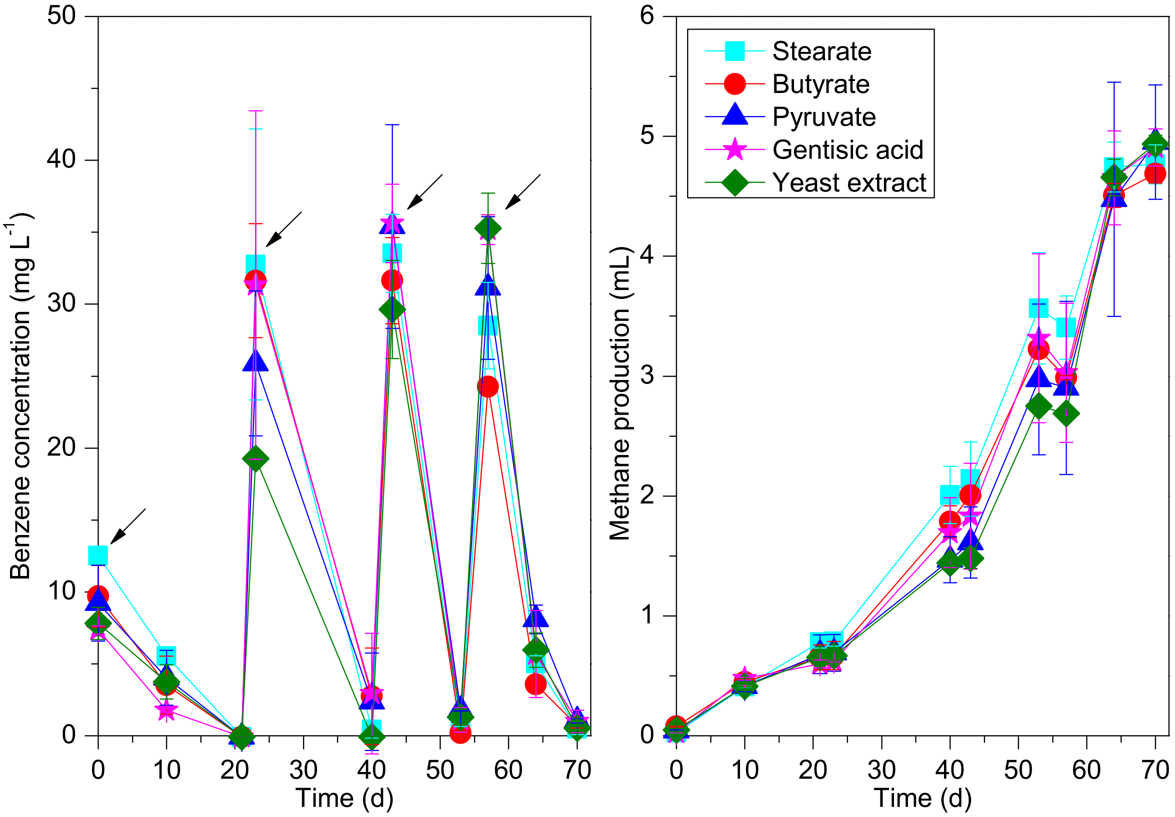


**Figure S4**: Effect of organic acid addition on benzene degradation and methane production in synthetic medium-based enrichment cultures. Each organic acid was separately added to the synthetic medium-based cultures at concentration of 0.01 g $L^{-1}$ and the cultures were named accordingly. Yeast extract cultures indicate the addition of yeast extract at concentration of 0.1 g $L^{-1}$. Error bars represent the standard deviation of duplicate samples. Black arrows indicate the times of benzene addition.

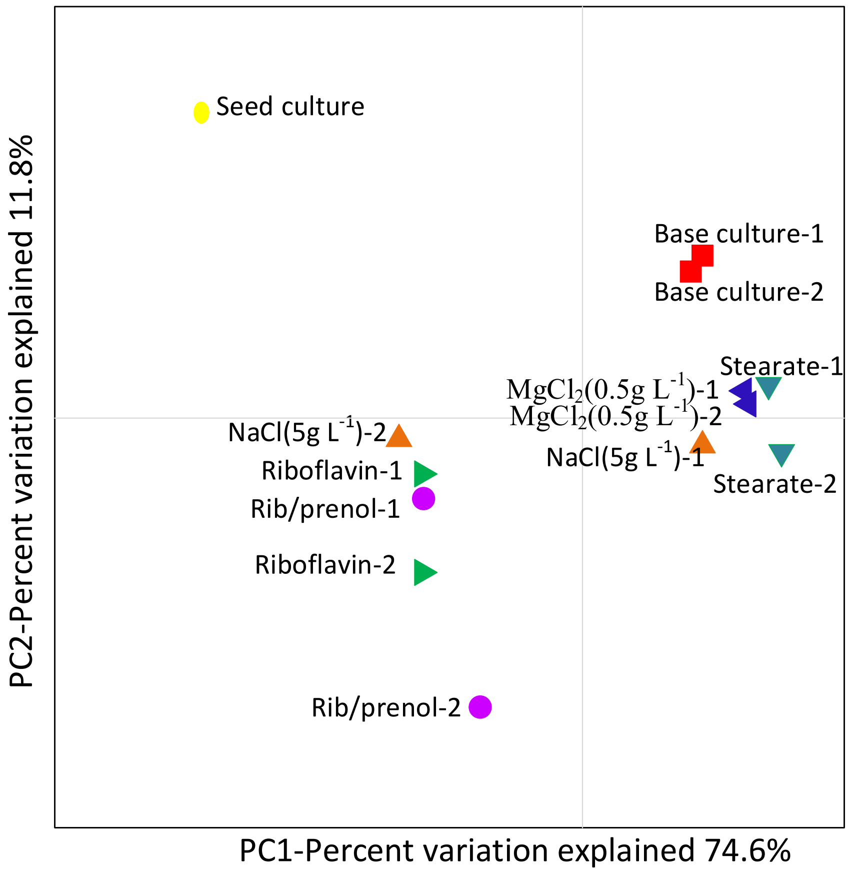


**Figure S5**: Principal coordinate analysis (PCoA) based on weighted UniFrac distance matrix of the microbial community between samples.

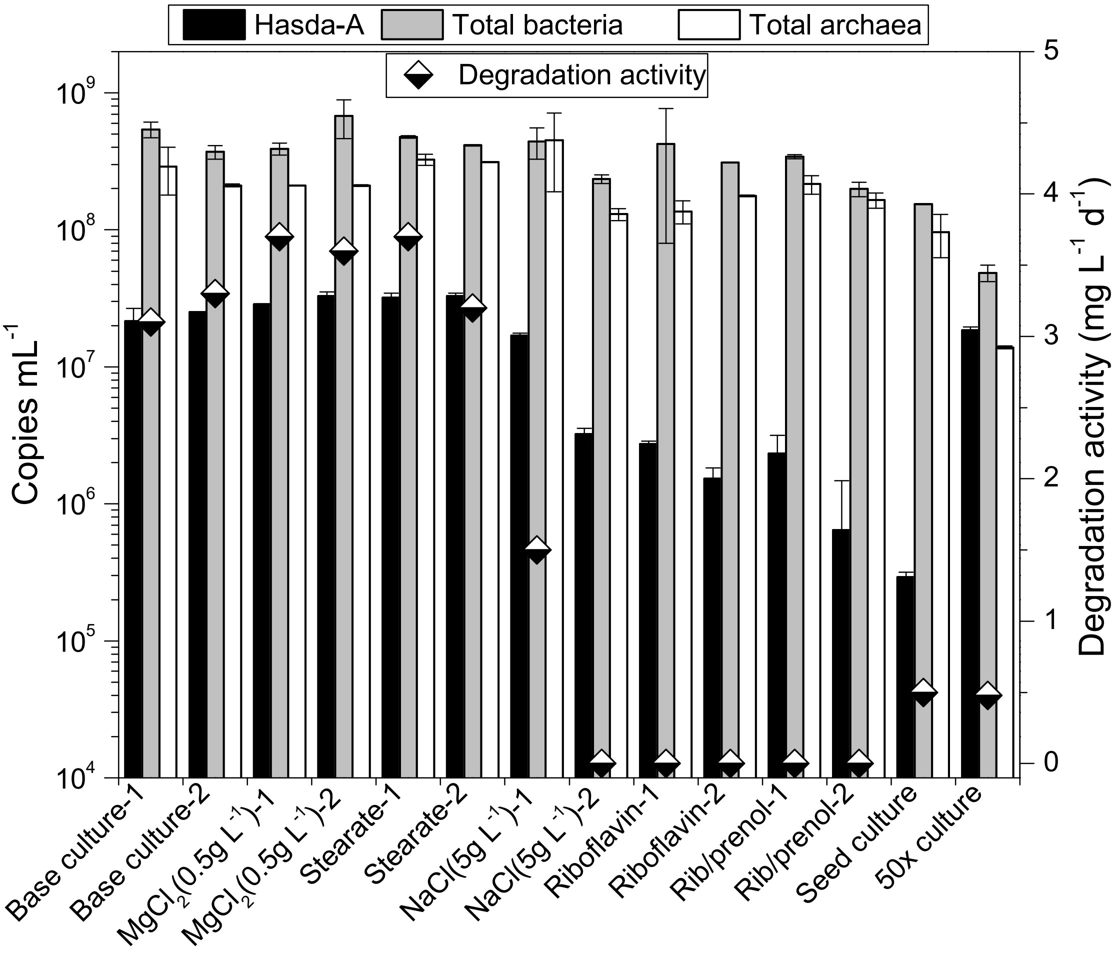


**Figure S6.** 16S rRNA gene copy numbers *vs.* benzene degradation activity. DNA samples of the enrichment cultures were the same as those used for community analysis by amplicon sequencing in **Fig. 2**, except for the "×50 culture" sample. Biomass samples of ×50 culture were collected on day 185. Error bars represent the standard deviation of triplicate qPCR assays.

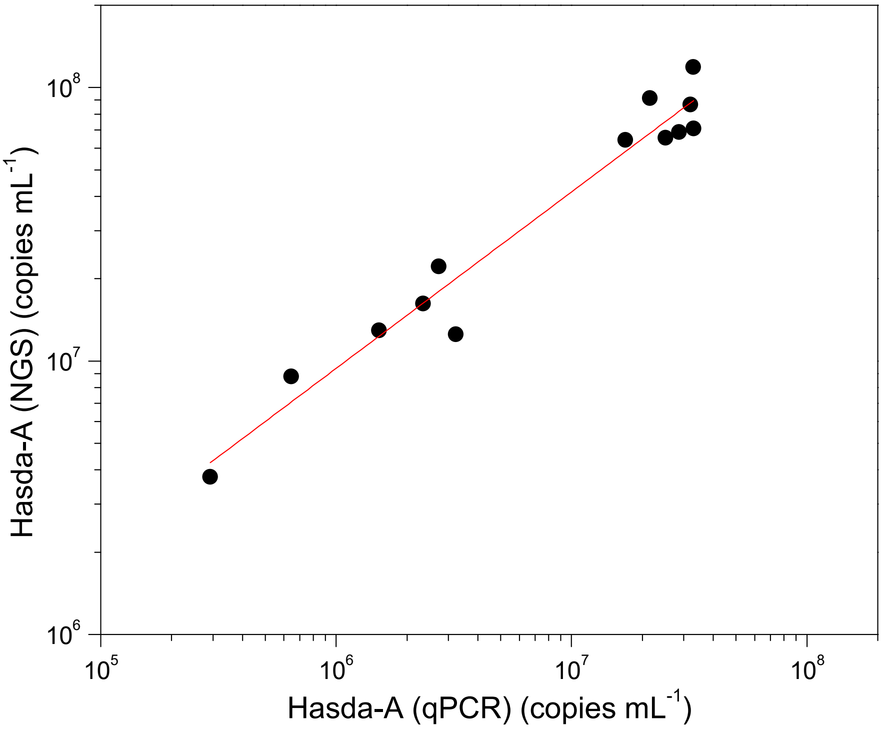


**Figure S7**: Correlation between the qPCR abundance and the estimated absolute abundance of Hasda-A. The estimated absolute abundance of Hasda-A was calculated by multiplying the relative abundances of Hasda-A obtained from next generation sequencing (NGS) with total bacteria quantified by qPCR. Pearson correlation of 0.98. The data is in log scale.

**Table S5**: Estimated doubling time of high-activity cultures (Base culture-1 and -2, $MgCl_2$(0.5g L$^{-1}$)-1 and 2, and stearate-1 and -2), designated as ×1 cultures. Data was calculated from **Fig. S6**.

| Organisms | ×1 cultures – Day 0 (dilution 3 times of seed culture) (copies mL$^{-1}$) | ×1 cultures – Day 68 (average of 6 high-activity cultures) (copies mL$^{-1}$) | Doubling time (Days)[a] |
|---|---|---|---|
| Hasda-A | $9.7 \times 10^4$ | $2.9 \times 10^7$ | 8.2 |
| Total Bacteria | $5.1 \times 10^7$ | $4.8 \times 10^8$ | 21.1 |
| Total Archaea | $3.2 \times 10^7$ | $2.6 \times 10^8$ | 22.5 |

*"a": Doubling time was estimated following the formula: $T_d = (T-T_0)/log_2(X/X_0)$; where $T_d$ is estimated doubling time in days; X and $X_0$ are biomass concentration (16S rRNA gene copy numbers per ml) at time T and $T_0$, respectively.*

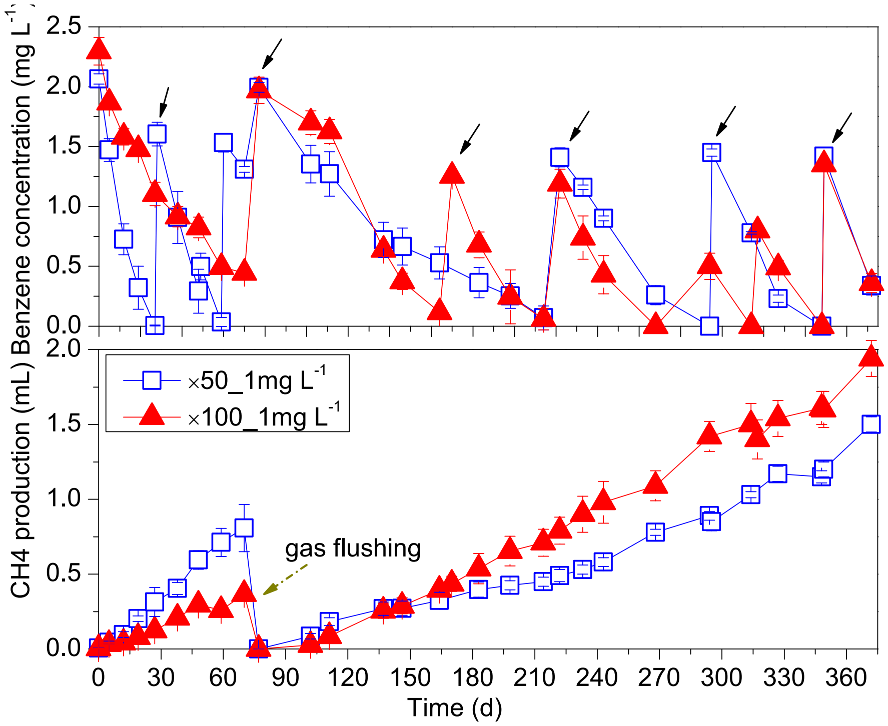


**Figure S8:** Benzene degradation and methane production in ×50 and ×100 diluted cultures at low benzene concentration. Error bars represent the standard deviation from triplicate samples. Each sample contains 200 mL culture in 500 mL bottle. Black solid arrows indicate the events of benzene addition.

**Table S6**: The relative abundance of the dominant OTUs (%) (average abundance in all samples > 0.4%) in 23 samples used for correlation analysis in **Fig. 4B, Fig. S9** and **Fig. S11**.

| OTUs* | Base culture | $MgCl_2$ (0.5g $L^{-1}$) | Stearate | NaCl (5g $L^{-1}$)-1 | NaCl (5g $L^{-1}$)-2 | Ribo flavin | Rib/ prenol | Seed | Tsu-Positive | Tsu-Negative | Enoki bashi | Enoki bashi $\alpha$ | Enoki bashi $\beta$ | IW-Enrich ment | SN-Enrich ment | TA-Enrich ment | TB-Enrich ment | TE-Enrich ment | IW-Sediment | SN-Sediment | TA-Sediment | TB-Sediment | TE-Sediment |
|---|---|---|---|---|---|---|---|---|---|---|---|---|---|---|---|---|---|---|---|---|---|---|---|
| (Crenarchaeota;c_MCG);s_ | 0.49 | 0.50 | 0.50 | 0.53 | 0.60 | 0.62 | 0.70 | 0.65 | 0.50 | 0.54 | 0.21 | 0.01 | 0.00 | 0.38 | 0.40 | 0.30 | 0.35 | 0.34 | 0.24 | 0.12 | 0.74 | 0.39 | 0.21 |
| (Crenarchaeota;c_MCG;o_pGrfC26);s_ | 1.55 | 1.76 | 1.74 | 1.93 | 2.21 | 2.26 | 2.32 | 2.55 | 0.67 | 0.90 | 0.28 | 0.01 | 0.00 | 1.88 | 2.05 | 2.14 | 2.06 | 2.17 | 1.50 | 0.79 | 2.40 | 1.48 | 1.94 |
| (Methanocellales);s_ | 1.24 | 1.22 | 1.29 | 1.35 | 1.45 | 1.32 | 1.25 | 1.20 | 0.28 | 0.27 | 0.00 | 0.00 | 0.00 | 0.03 | 0.05 | 0.11 | 0.03 | 0.05 | 0.01 | 0.01 | 0.03 | 0.07 | 0.01 |
| (Candidatus Methanoregula);s_ | 4.84 | 5.05 | 5.00 | 3.97 | 2.94 | 2.96 | 3.39 | 2.49 | 1.03 | 0.59 | 2.07 | 1.34 | 0.53 | 0.11 | 0.09 | 0.09 | 0.12 | 0.13 | 0.08 | 0.05 | 0.11 | 0.09 | 0.08 |
| (Methanosaeta);s_ | 5.17 | 5.18 | 5.29 | 5.13 | 3.90 | 3.64 | 3.98 | 2.17 | 1.08 | 0.65 | 0.71 | 0.54 | 0.30 | 0.41 | 0.47 | 0.46 | 0.47 | 0.47 | 0.17 | 0.13 | 0.30 | 0.33 | 0.29 |
| (Thermoplasmata;o_E2;f_DHVEG-1);g_;s_ | 3.42 | 4.03 | 4.12 | 4.86 | 5.15 | 5.87 | 6.18 | 4.45 | 0.59 | 0.92 | 0.72 | 0.01 | 0.02 | 1.36 | 1.40 | 1.25 | 1.46 | 1.44 | 1.37 | 0.60 | 2.25 | 1.44 | 0.71 |
| (k_Bacteria);_ | 1.36 | 1.42 | 1.31 | 1.31 | 1.60 | 1.76 | 1.75 | 1.62 | 0.67 | 0.83 | 0.74 | 1.00 | 0.48 | 0.42 | 0.31 | 0.50 | 0.48 | 0.54 | 0.34 | 0.18 | 0.58 | 0.53 | 0.26 |
| (k_Bacteria);p_;s_ | 0.75 | 0.72 | 0.72 | 0.64 | 0.71 | 0.71 | 0.73 | 0.76 | 0.84 | 0.92 | 1.18 | 0.29 | 0.32 | 0.36 | 0.36 | 0.44 | 0.52 | 0.45 | 0.47 | 0.56 | 0.73 | 0.60 | 0.38 |
| (Bacteria;p_AC1;c_SHA-114);s_ | 0.77 | 0.78 | 0.73 | 0.77 | 0.96 | 0.94 | 0.90 | 1.10 | 0.26 | 0.37 | 0.57 | 0.18 | 0.10 | 0.33 | 0.43 | 0.52 | 0.37 | 0.43 | 0.16 | 0.12 | 0.41 | 0.37 | 0.18 |
| (Acidobacteria-6;o_CCU21);s_ | 0.37 | 0.25 | 0.29 | 0.29 | 0.33 | 0.40 | 0.38 | 0.49 | 0.73 | 0.76 | 0.07 | 0.01 | 0.01 | 1.11 | 1.07 | 1.04 | 0.96 | 1.30 | 1.33 | 1.03 | 0.99 | 0.99 | 1.51 |
| (Acidobacteria;c_BPC102;o_MVS-40)s_ | 0.41 | 0.41 | 0.44 | 0.40 | 0.42 | 0.48 | 0.46 | 0.54 | 0.53 | 0.70 | 0.20 | 0.01 | 0.01 | 1.03 | 0.94 | 1.06 | 1.08 | 1.22 | 1.10 | 1.01 | 1.25 | 1.26 | 1.71 |
| (Acidobacteria;c_OS-K);s_ | 1.37 | 1.10 | 1.08 | 1.27 | 1.44 | 1.44 | 1.33 | 1.61 | 0.59 | 0.54 | 0.14 | 0.03 | 0.03 | 0.25 | 0.13 | 0.16 | 0.19 | 0.27 | 0.21 | 0.35 | 0.24 | 0.30 | 0.19 |
| (Gaiellaceae);s_ | 0.10 | 0.09 | 0.10 | 0.14 | 0.16 | 0.13 | 0.17 | 0.21 | 1.69 | 1.71 | 0.00 | 0.00 | 0.01 | 0.76 | 0.59 | 0.54 | 0.58 | 0.65 | 0.38 | 0.35 | 0.29 | 0.28 | 0.51 |
| (Bacteroidales);s_ | 1.30 | 1.61 | 1.67 | 1.85 | 1.84 | 1.59 | 1.69 | 2.06 | 0.67 | 1.01 | 2.03 | 0.38 | 0.86 | 4.76 | 5.23 | 4.53 | 4.17 | 4.62 | 4.71 | 3.68 | 4.24 | 5.06 | 3.57 |
| (Chlorobi;c_BSV26;o_PK329);s_ | 2.61 | 2.70 | 2.68 | 2.77 | 3.62 | 2.99 | 2.76 | 2.39 | 0.93 | 1.06 | 1.01 | 0.04 | 0.03 | 0.26 | 0.33 | 0.31 | 0.34 | 0.29 | 0.52 | 0.46 | 0.50 | 0.31 | 0.34 |
| (Anaerolineae);s_ | 1.28 | 1.14 | 1.18 | 1.05 | 1.23 | 1.13 | 1.11 | 1.63 | 0.81 | 0.87 | 0.19 | 0.01 | 0.01 | 0.14 | 0.21 | 0.12 | 0.23 | 0.19 | 0.16 | 0.24 | 0.22 | 0.18 | 0.10 |
| (Anaerolineae;o_GCA004);s_ | 5.29 | 5.19 | 4.89 | 5.68 | 7.06 | 6.89 | 6.52 | 7.67 | 3.13 | 3.37 | 1.16 | 0.46 | 0.31 | 0.79 | 0.83 | 0.77 | 0.79 | 0.76 | 0.64 | 0.40 | 0.78 | 0.72 | 0.53 |
| (Anaerolineae;o_SHA-20);s_ | 3.56 | 3.35 | 3.03 | 3.21 | 3.90 | 3.67 | 3.74 | 5.58 | 1.05 | 1.03 | 0.58 | 0.07 | 0.05 | 0.82 | 0.59 | 1.88 | 1.26 | 0.94 | 0.45 | 0.24 | 1.29 | 0.88 | 0.28 |
| (Anaerolineae;o_SJA-15);s_ | 0.90 | 0.71 | 0.67 | 0.70 | 0.87 | 0.94 | 0.87 | 1.14 | 0.75 | 0.65 | 0.12 | 0.03 | 0.02 | 0.52 | 0.23 | 0.44 | 0.44 | 0.41 | 0.39 | 0.25 | 0.48 | 0.50 | 0.25 |
| (Anaerolineae;o_envOPS12);s_ | 3.75 | 3.54 | 3.63 | 3.65 | 4.33 | 4.62 | 4.54 | 4.94 | 1.41 | 1.37 | 0.55 | 0.13 | 0.18 | 0.77 | 0.44 | 0.65 | 0.64 | 0.71 | 0.67 | 0.95 | 0.73 | 0.86 | 0.85 |
| (Ellin6529);s_ | 0.07 | 0.07 | 0.07 | 0.06 | 0.08 | 0.11 | 0.11 | 0.15 | 0.59 | 0.64 | 0.01 | 0.00 | 0.00 | 1.29 | 1.21 | 1.17 | 1.21 | 1.73 | 1.39 | 1.63 | 1.28 | 1.24 | 1.83 |
| (Planococcaceae);_ | 0.09 | 0.09 | 0.08 | 0.07 | 0.13 | 0.11 | 0.10 | 0.19 | 0.54 | 0.59 | 0.03 | 0.04 | 0.03 | 19.54 | 18.15 | 16.87 | 19.21 | 16.57 | 12.50 | 6.38 | 8.40 | 13.46 | 9.26 |
| (Clostridium);s_ | 0.79 | 0.81 | 0.81 | 0.96 | 1.13 | 1.21 | 1.23 | 1.57 | 0.34 | 0.38 | 0.02 | 0.02 | 0.05 | 1.55 | 1.31 | 1.33 | 1.28 | 1.39 | 1.24 | 0.68 | 0.88 | 1.03 | 0.75 |
| (Clostridium);s_bowmanii | 0.19 | 0.16 | 0.17 | 0.26 | 0.25 | 0.24 | 0.25 | 0.39 | 0.11 | 0.15 | 0.00 | 0.00 | 0.00 | 2.16 | 2.38 | 2.13 | 2.33 | 1.95 | 1.64 | 0.92 | 1.22 | 1.21 | 1.16 |
| (Veillonellaceae);s_ | 0.02 | 0.01 | 0.02 | 0.00 | 0.02 | 0.02 | 0.02 | 0.02 | 0.00 | 0.00 | 0.00 | 0.01 | 0.03 | 2.06 | 1.94 | 1.60 | 1.64 | 1.77 | 1.50 | 0.67 | 0.97 | 0.71 | 1.04 |
| (Fusibacter);s_ | 0.69 | 0.60 | 0.63 | 0.71 | 0.97 | 0.92 | 0.95 | 1.08 | 0.20 | 0.17 | 0.36 | 0.05 | 1.99 | 16.77 | 18.16 | 15.81 | 15.94 | 15.16 | 12.86 | 5.93 | 7.74 | 8.37 | 9.01 |
| (Gemmatimonadetes;c_Gemm-1);s_ | 0.09 | 0.10 | 0.09 | 0.08 | 0.10 | 0.09 | 0.10 | 0.12 | 0.56 | 0.63 | 0.46 | 0.02 | 0.02 | 0.72 | 0.79 | 0.66 | 0.51 | 0.63 | 0.69 | 0.90 | 0.46 | 0.65 | 2.06 |
| ([Thermodesulfovibrionaceae]);s_ | 0.12 | 0.14 | 0.14 | 0.15 | 0.17 | 0.18 | 0.19 | 0.17 | 2.83 | 1.93 | 2.18 | 3.93 | 11.46 | 0.09 | 0.06 | 0.07 | 0.07 | 0.04 | 0.11 | 0.05 | 0.04 | 0.10 | 0.10 |
| ([Thermodesulfovibrionaceae];g_GOUTA19);s_ | 0.32 | 0.33 | 0.36 | 0.31 | 0.53 | 0.50 | 0.49 | 0.40 | 1.47 | 1.63 | 3.61 | 0.07 | 0.03 | 0.30 | 0.37 | 0.26 | 0.31 | 0.29 | 0.27 | 0.31 | 0.18 | 0.29 | 0.45 |
| (p_OD1);s_ | 1.61 | 1.81 | 2.80 | 4.27 | 1.04 | 1.35 | 1.29 | 1.08 | 0.78 | 0.76 | 1.10 | 1.00 | 0.73 | 0.32 | 0.35 | 0.47 | 0.41 | 0.38 | 0.26 | 0.13 | 0.24 | 0.36 | 0.19 |
| (p_OP8;c_OP8_1);s_ | 1.51 | 1.53 | 1.34 | 1.43 | 1.83 | 1.78 | 1.72 | 2.35 | 0.88 | 1.00 | 0.27 | 0.07 | 0.05 | 0.74 | 0.69 | 1.28 | 1.27 | 0.99 | 0.51 | 0.43 | 1.27 | 1.04 | 0.39 |
| (p_OP8;c_OP8_1;o_SHA-124);s_ | 0.64 | 0.62 | 0.64 | 0.71 | 0.90 | 0.83 | 0.80 | 0.76 | 0.45 | 0.56 | 0.93 | 0.09 | 0.05 | 0.86 | 0.85 | 1.03 | 0.91 | 0.98 | 1.41 | 0.51 | 1.87 | 1.22 | 0.83 |
| (Pirellulaceae);s_ | 0.35 | 0.30 | 0.30 | 0.27 | 0.30 | 0.32 | 0.35 | 0.37 | 0.21 | 0.23 | 0.03 | 0.01 | 0.01 | 0.74 | 0.83 | 0.86 | 0.83 | 0.85 | 0.87 | 0.99 | 0.74 | 0.85 | 0.64 |
| (Hyphomicrobium);s_ | 0.25 | 0.18 | 0.19 | 0.20 | 0.31 | 0.31 | 0.32 | 0.35 | 0.23 | 0.23 | 0.01 | 0.01 | 0.43 | 1.01 | 1.16 | 0.78 | 1.00 | 1.02 | 0.82 | 0.51 | 0.59 | 0.51 | 0.57 |
| (Betaproteobacteria);s_ | 0.11 | 0.13 | 0.09 | 0.15 | 0.20 | 0.11 | 0.09 | 0.04 | 1.08 | 0.83 | 0.04 | 0.03 | 0.02 | 0.90 | 0.87 | 0.75 | 0.84 | 0.85 | 0.94 | 0.67 | 0.53 | 0.66 | 2.62 |
| (Methylophilales);s_ | 0.13 | 0.14 | 0.08 | 0.14 | 0.19 | 0.09 | 0.04 | 0.01 | 0.03 | 0.03 | 1.10 | 10.73 | 8.97 | 0.00 | 0.00 | 0.00 | 0.00 | 0.01 | 0.09 | 0.15 | 0.05 | 0.08 | 0.04 |
| (Rhodocyclaceae;g_Dok59);s_ | 0.29 | 0.19 | 0.25 | 0.13 | 0.10 | 0.31 | 0.28 | 0.06 | 0.78 | 0.56 | 13.24 | 27.95 | 15.79 | 0.03 | 0.05 | 0.04 | 0.04 | 0.04 | 0.24 | 1.11 | 0.06 | 0.07 | 0.07 |
| (Deltaproteobacteria)Hasda-A | 17.30 | 17.56 | 17.55 | 14.59 | 5.33 | 4.78 | 4.58 | 2.45 | 11.15 | 5.15 | 6.15 | 4.30 | 2.38 | 1.63 | 1.56 | 2.09 | 1.34 | 1.91 | 1.69 | 1.24 | 2.46 | 1.52 | 1.29 |
| (Deltaproteobacteria;o_BPC076);s_ | 2.93 | 2.86 | 2.90 | 3.17 | 4.09 | 4.00 | 4.21 | 3.77 | 4.29 | 4.61 | 1.09 | 0.02 | 0.03 | 1.90 | 2.05 | 1.80 | 1.71 | 2.17 | 1.48 | 0.92 | 2.22 | 1.83 | 1.39 |
| (Desulfarculaceae);s_ | 0.36 | 0.36 | 0.30 | 0.42 | 0.46 | 0.26 | 0.29 | 0.26 | 0.48 | 0.54 | 0.81 | 0.05 | 0.03 | 0.37 | 0.41 | 0.41 | 0.34 | 0.42 | 0.53 | 0.31 | 1.08 | 0.69 | 0.44 |
| (Geobacter);s_ | 0.07 | 0.05 | 0.06 | 0.06 | 0.10 | 0.15 | 0.46 | 0.09 | 0.59 | 0.76 | 8.92 | 3.84 | 3.05 | 1.96 | 1.95 | 2.51 | 1.42 | 2.28 | 1.97 | 1.87 | 2.17 | 1.75 | 1.27 |
| (Myxococcales);s_ | 0.41 | 0.45 | 0.34 | 0.29 | 0.32 | 0.36 | 0.34 | 0.54 | 2.16 | 1.79 | 0.66 | 0.00 | 0.01 | 0.31 | 0.39 | 0.39 | 0.29 | 0.34 | 0.54 | 0.48 | 0.49 | 0.64 | 1.76 |
| (Syntrophaceae);s_ | 0.90 | 0.92 | 0.86 | 0.88 | 1.13 | 1.16 | 1.35 | 1.13 | 0.37 | 0.47 | 1.81 | 3.39 | 2.04 | 0.33 | 0.42 | 0.41 | 0.31 | 0.42 | 0.61 | 0.43 | 0.83 | 0.47 | 0.49 |
| (Desulfobacca);s_ | 1.83 | 1.61 | 1.49 | 1.61 | 2.19 | 2.34 | 2.21 | 1.56 | 4.66 | 5.16 | 0.12 | 0.30 | 0.46 | 0.27 | 0.21 | 0.34 | 0.26 | 0.35 | 0.18 | 0.29 | 0.40 | 0.52 | 0.18 |
| (Syntrophus);s_aciditrophicus | 1.03 | 0.94 | 0.97 | 1.00 | 1.11 | 1.37 | 1.37 | 1.40 | 0.01 | 0.01 | 0.00 | 0.00 | 0.00 | 0.01 | 0.00 | 0.02 | 0.02 | 0.02 | 0.01 | 0.02 | 0.03 | 0.02 | 0.00 |
| (Methylobacter);s_luteus | 0.32 | 0.22 | 0.32 | 0.46 | 0.25 | 0.34 | 0.32 | 0.66 | 0.99 | 1.51 | 1.42 | 8.66 | 12.85 | 0.00 | 0.02 | 0.01 | 0.01 | 0.00 | 0.01 | 0.03 | 0.01 | 0.00 | 0.00 |
| (Pseudomonas);_ | 0.01 | 0.01 | 0.02 | 0.02 | 0.01 | 0.03 | 0.03 | 0.04 | 0.11 | 0.14 | 1.49 | 3.66 | 3.79 | 0.12 | 0.01 | 0.02 | 0.01 | 0.00 | 0.02 | 0.01 | 0.03 | 0.04 | 0.01 |
| (Spirochaetaceae);s_ | 1.70 | 1.63 | 1.49 | 1.68 | 2.28 | 2.35 | 2.25 | 2.61 | 0.66 | 0.94 | 0.95 | 0.02 | 0.01 | 1.30 | 1.32 | 1.36 | 1.27 | 1.34 | 1.27 | 1.16 | 1.39 | 1.17 | 1.41 |
| (Treponema);s_ | 0.00 | 0.00 | 0.00 | 0.00 | 0.00 | 0.01 | 0.01 | 0.00 | 0.03 | 0.02 | 6.83 | 5.62 | 4.89 | 0.19 | 0.10 | 0.18 | 0.20 | 0.15 | 0.18 | 0.11 | 0.15 | 0.28 | 0.16 |

* The OTUs were annotated at the lowest level of known taxonomic classification. The "p_", "c_", "o_", "f_", "g_" and "s_" symbols indicate the taxonomic ranks at phylum, class, order, family, genus, and species, respectively. The symbol "s_" without taxonomic names at the end indicates the lack of species-level annotation in reference database.

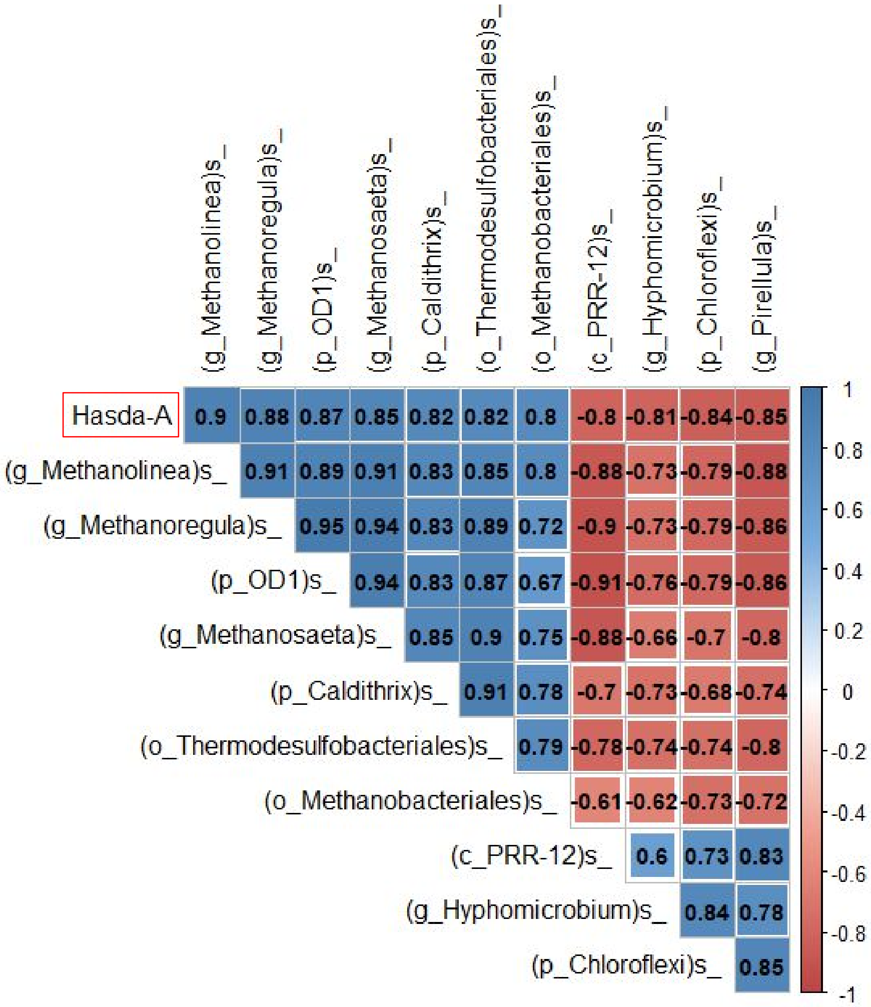


**Figure S9**: A Spearman correlation matrix of microbial phylotypes having a strong correlation with *Deltaproteobacterium* Hasda-A (coefficient > 0.8 & *p-value* < 0.0001). Data were filtered to keep only population that were, on average, have more than 2 (total count > 46) reads per sample and presence in 30% of samples (6 samples). The relative abundance was calculated by dividing each microbial population by the total counts of the sample. The "*p*_", "*c*_", "*o*_", "*f*_", "*g*_", and "*s*_" symbols indicate the taxonomic ranks at phylum, class, order, family, genus, and species, respectively.  The symbol "*s*_" without taxonomic names at the end indicates the lack of species-level annotation in reference database.

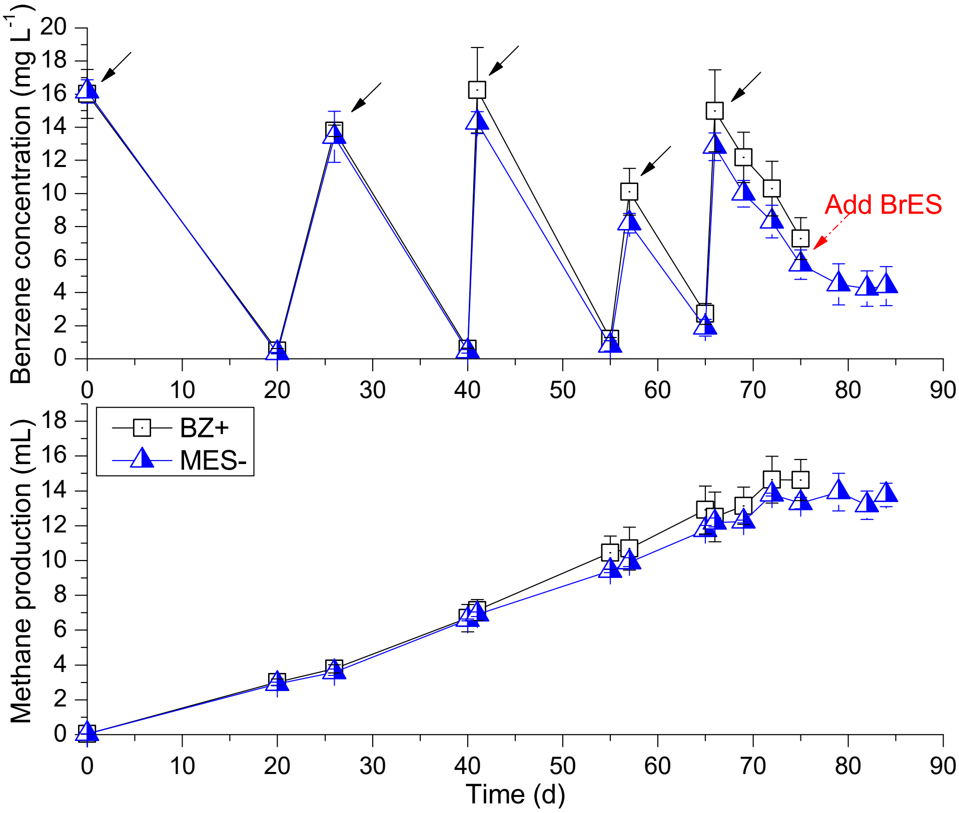


**Figure S10:** Benzene concentration and methane production in inhibition experiments. **"BZ+"**: Positive benzene degradation; **"MES-"**: inhibition of methanogenesis by 2-Bromoethanesulfonate (BrES). ×10 diluted cultures were setup (200 mL culture in 500 mL vials) in triplicate for each experiment. On day 75, biomass samples from "BZ+" were collected while BrES (final conc. of 5 mM) were added into "MES-" cultures. BrES addition inhibited benzene degradation and methane production completely within 6 d. Black arrows indicated the event of benzene addition.

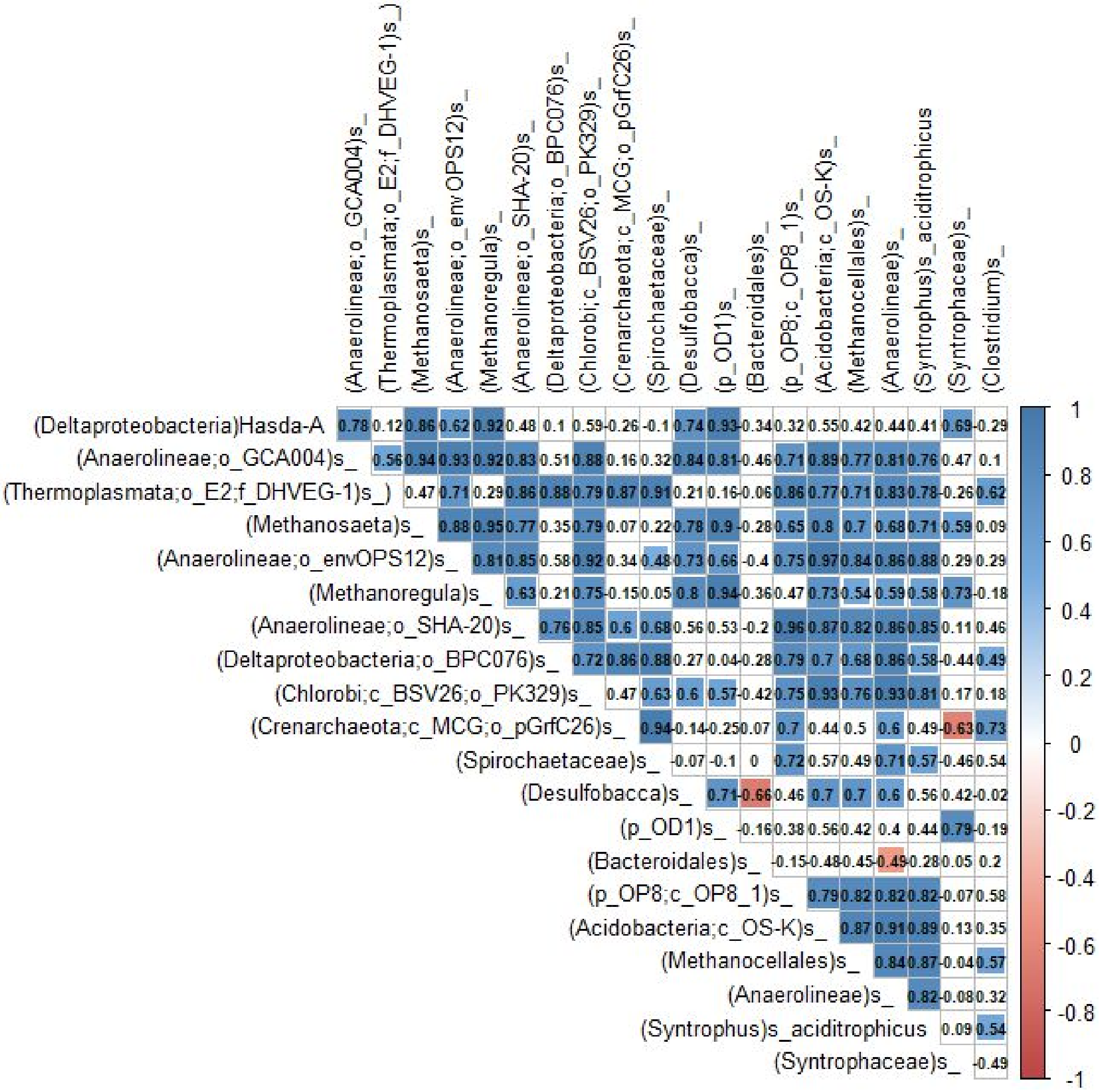


**Figure S11**: SparCC correlation matrix of the abundant microbial populations presented in **Fig. 2B**. The value of the pairwise correlation coefficient is shown in each box of the grid. The correlation with two-sided pseudo *p-values* > 0.0001 (based on bootstrapping of 100 repetitions) are considered as insignificant and left blank. The order and annotation of the microbial population was presented as in **Fig. 2B**.

**References**

BENGTSON, P., STERNGREN, A. E. & ROUSK, J. 2012. Archaeal Abundance across a pH Gradient in an Arable Soil and Its Relationship to Bacterial and Fungal Growth Rates. *Appl Environ Microbiol,* 78**,** 5906.

KASUGA, I., NAKAGAKI, H., KURISU, F. & FURUMAI, H. 2010. Predominance of ammonia-oxidizing archaea on granular activated carbon used in a full-scale advanced drinking water treatment plant. *Water Research,* 44**,** 5039-5049.

NOGUCHI, M., KURISU, F., SEKIGUCHI, Y., KASUGA, I. & FURUMAI, H. 2016. Microbial community structure of methanogenic benzene-degrading cultures enriched from five different sediments. *The Journal of General and Applied Microbiology,* 62**,** 266-271.

SAKAI, N., KURISU, F., YAGI, O., NAKAJIMA, F. & YAMAMOTO, K. 2009. Identification of putative benzene-degrading bacteria in methanogenic enrichment cultures. *JBB,* 108**,** 501-507.
